# Metal(triphenylphosphine)-atovaquone Complexes: Synthesis, Antimalarial Activity, and Suppression of Heme Detoxification

**DOI:** 10.1021/acs.inorgchem.4c02751

**Published:** 2024-08-26

**Authors:** Luana Daniel, Arquímedes Karam, Chris Hebert J. Franco, Camila Conde, Adrielle Sacramento de Morais, Joel Mosnier, Isabelle Fonta, Wilmer Villarreal, Bruno Pradines, Diogo Rodrigo M. Moreira, Maribel Navarro

**Affiliations:** † Laboratório de Química Bioinorgânica e Catalise, Departamento Química, Instituto de Ciências Exatas, Universidade Federal de Juiz de Fora, Juiz de Fora, Minas Gerais 36036-900, Brazil; ‡ Centro de Química Estrutural, Institute of Molecular Sciences, Instituto Superior Técnico, Universidade de Lisboa, Lisbon, 1049-001, Portugal; § Instituto Gonçalo Moniz, FIOCRUZ, Salvador, Bahia 40296-710, Brazil; ∥ Unité Parasitologie et Entomologie, Institut de Recherche Biomédicale des Armées, Marseille, 13005, France; ⊥ Aix-Marseille Univ, SSA, AP-HM, RITMES, Marseille, 13005, France; # IHU Méditerranée Infection, 19-21 Boulevard Jean Moulin, Marseille, 13005, France; ∇ Centre National de Référence du Paludisme, Marseille, 13005, France; ○ Grupo de Química Inorgânica Medicinal e Reações Aplicadas, Instituto de Química, Universidade Federal do Rio Grande do Sul, Porto Alegre, Rio Grande do Sul 91501-970, Brazil

## Abstract

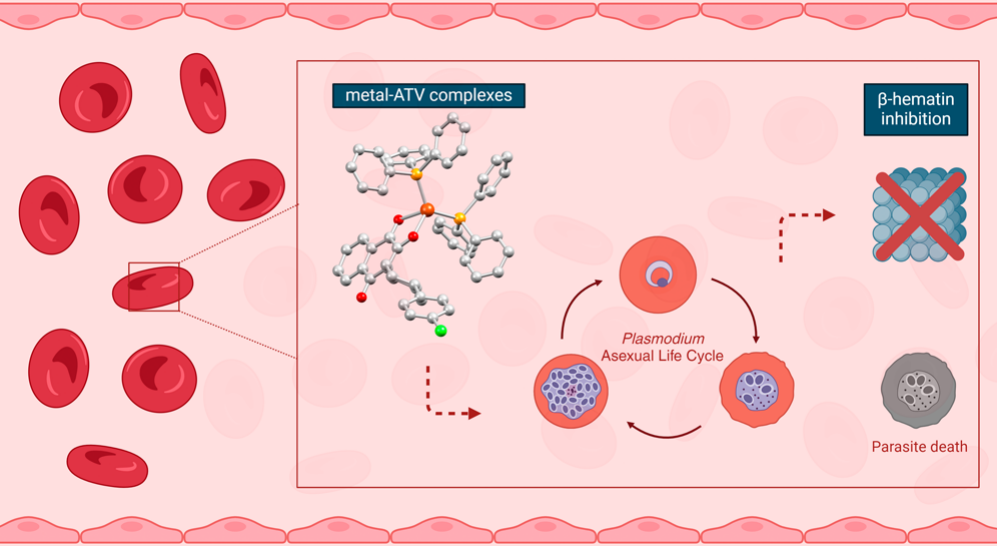

To ascertain the bioinorganic chemistry of metals conjugated with quinones, the complexes [Ag(ATV)(PPh_3_)_2_] (**1**), [Au(ATV)(PPh_3_)]·2H_2_O (**2**), and [Cu(ATV)(PPh_3_)_2_] (**3**) were synthesized by the coordination of the antimalarial naphthoquinone atovaquone (ATV) to the starting materials [Ag(PPh_3)2_]NO_3_, [Au(PPh_3_)Cl], and [Cu(PPh_3_)_2_NO_3_], respectively. These complexes were characterized by analytical and spectroscopical techniques. X-ray diffraction of single crystals precisely confirmed the coordination mode of ATV to the metals, which was monodentate or bidentate, depending on the metal center. Both coordination modes showed high stability in the solid state and in solution. All three complexes showed negative log *D* values at pH 5, but at pH 7.4, while complex **2** continued to have a negative log *D* value, complexes **1** and **3** displayed positive values, indicating a more hydrophilic character. ATV and complexes **1**–**3** could bind to ferriprotoporphyrin IX (FePPIX); however, only complexes **1**–**3** could inhibit β-hematin crystal formation. Phenotype-based activity revealed that all three metal complexes are able to inhibit the growth of *P. falciparum* with potency and selectivity comparable to those of ATV, while the starting materials lack this activity. The outcomes of this chemical design may provide significant insights into structure–activity relationships for the development of new antimalarial agents.

## Abstract 2

The synthesis of three new coordination complexes was achieved using the antimalarial naphthoquinone atovaquone as a ligand. The complexes [Ag(ATV)(PPh_3_)_2_] (**1**), [Au(ATV)(PPh_3_)]·2H_2_O (**2**), and [Cu(ATV)(PPh_3_)_2_] (**3**) were characterized in the solid state and in solution; single-crystal X-ray diffraction was solved. Structure−activity relationship annotation supports the idea that antimalarial activity of atovaquone depends on quinone redox status and that metal coordination can alter the molecular and cell-based interactions.

## Introduction

Malaria, caused by the pathogen Plasmodium, is a major global health issue. In 2022, there were an estimated 249 million cases, resulting in 608,000 deaths worldwide.^1^ According to the World Health Organization, Africa is the continent most impacted by malaria infection, accounting for 95% of deaths worldwide, mostly young children.^1^ To eradicate this infectious disease, new antiparasitic drugs are needed, especially ones that are active against multiple stages of the parasite’s life cycle and also against drug-resistant strains.^2^ One masterful evolutionary adaptation of Plasmodium is its reliance on several mitochondrial processes not only to provide adenosine triphosphate, but also for the biosynthesis of orotate, a precursor of pyrimidine that is essential for the biosynthesis of nucleic acids.^3−5^ The disruption of these mitochondrial processes is detrimental to the growth and survival of the parasite, and this is the main mode of action of the antimalarial naphthoquinone atovaquone (ATV). It slows down the growth of the parasites by binding to the cytochrome bc1 complex (ubiquinol: cytochrome *c* oxidoreductase, respiratory complex III), affecting the recycling of the ubiquinol-to-ubiquinone redox status and ultimately causing collapse of mitochondrial membrane potential in the parasite cells.^6,7^

ATV has been used in malaria treatment for many decades and remains a key component of the therapy,^8^ especially because of its unique mechanism of action, its long-lasting effect on asexual blood parasite stages, and its great potential to block the transmission of the parasite from mosquito vectors to humans.^9^ The main limitations of ATV are its slow-acting effect on the inhibition of parasite growth,^10^ incomplete eradication of late-stage parasites (trophozoites and schizonts),^11^ and the emerging resistance of the parasite to ATV in comparison with other therapies.^12^ Indeed, atovaquone-proguanil treatment failures have been reported in Africa,^13,14^ which were associated with a mutation on codon 268 of the cytochrome *b* gene in parasites at a late stage of treatment.^12,13^ This mutation has not been detected in parasites from untreated patients or from samples collected in early treatment.^14,15^ One way to counteract these limitations is to improve the antiplasmodial activity of ATV by developing new derivatives. Several studies have shown that the 1,2-dioxyal component of ATV is a structural determinant for ATV’s binding to the cytochrome *b* at the ubiquinone binding site.^5−7^

In terms of drug design, there are at least two approaches for overcoming the limitations of ATV. The first is to explore quinone reactivity as a redox-active motif. Quinone variants are more likely to undergo a bioreductive step that would attack not only complex III, but also affect the redox homeostasis of parasite flavoenzymes.^16−18^ This strategy has delivered excellent results in terms of potency, effectiveness, and overcoming the slowness of action,^19^ but it has often come with the emergence of an imbalance in the redox homeostasis of the flavoenzymes, which is the main mechanism responsible for antiplasmodial activity.^20^ A second approach involves creating a quinone delivery system by means of metal-quinone complexes, where the dissociation of quinone from the metal complex occurs in a bio reductive step, resulting in the generation of semiquinone, quinone, and metal fragments with different types of activity.^21−23^ This approach has been used for several quinones in the context of other infectious diseases, but not yet for ATV and malaria. The dissociation of quinone from a metal can vary structurally and kinematically depending on the metal and the vicinity of the auxiliary ligand,^24−26^ and no obvious mechanistic model is currently available.

The coordination of antimalarial drugs such as the 4-aminoquinolines chloroquine or amodiaquine to transition metals has been shown to significantly enhance the potency and efficacy of antimalarial properties and, more importantly, increase the spectrum of action against multiple stages of the *Plasmodium* life cycle.^27−30^ This broader spectrum of activity is explained by the fact that these metallodrugs can interact with multiple parasite targets, while metal-free quinolines typically cannot.^27^ We believed this design concept may have the potential to be employed for the synthesis of metal–quinone complexes. However, less obvious is the rational choice of transition metal for such complexes.

Gold(I) complexes have a long history in modern medicinal chemistry and gold complexes with quinolines have proved to be antimalarial agents.^31−33^ The same is true for copper(I) complexes.^33,34^ In contrast, the coordination between silver(I) and antimalarial drugs is relatively scarce, but there are two prior reports showing that silver(I) complexes containing phosphine and thiazolidine ligands may have remarkable inhibitory activity against chloroquine-resistant *P. falciparum*.^33,35^

In this work, we describe the coordination of ATV to gold(I), silver(I), and copper(I) ions that contain a phosphine molecule as an auxiliary ligand. These metal–ATV complexes were synthesized and fully characterized in solid state and in solution. In addition, the antimalarial activity of the metal–ATV complexes was evaluated to determine their ability to inhibit the growth of *P. falciparum*. It is suggested that the [M(PPh_3_)(ATV)] complex may deliver ATV inside the parasite cells, thereby promoting collapse of mitochondrial membrane potential, while the metal fragments could act on other essential targets of the parasites. In light of this, we demonstrated the inhibition of the heme detoxification pathway as a potential mechanism of action for these metal–ATV complexes.

## Results and Discussion

### Synthesis and Chemical Characterization

**Scheme 1:**
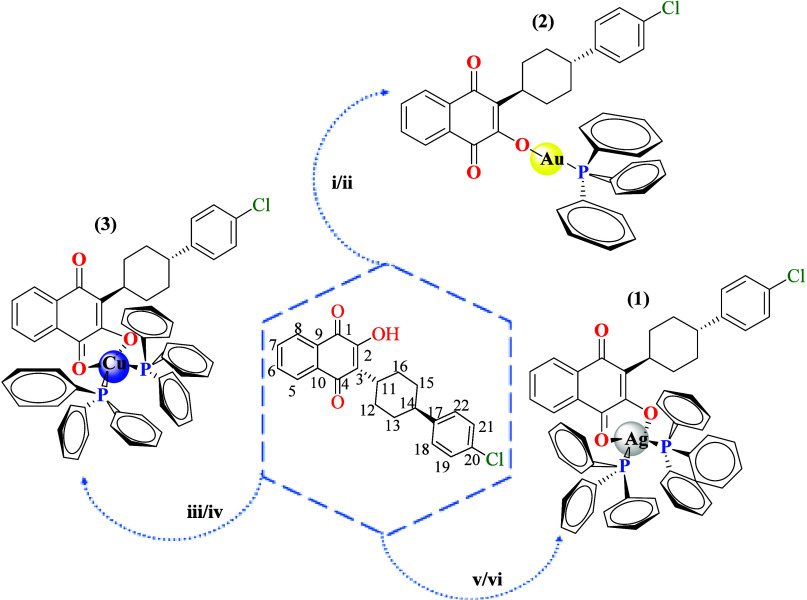
Synthesis of M(ATV)(PPh_3_) Complexes, Where M = silver(I), gold(I), and copper(I) (i) [Au(PPh_3_)_2_Cl]/AgNO_3_/CH_2_Cl_2_/MeOH, room temperature (RT), 24 h; (ii) NaOCH_3_/CH_2_Cl_2,_ RT, 3 h; (iii) KOH/MeOH, RT, 1 h; (iv) [Cu(PPh_3_)_2_NO_3_]/CH_2_Cl_2_, RT, 3 h; (v) KOH/MeOH, RT, 1 h; (vi) [Ag(PPh_3_)_2_NO_3_]/CH_2_Cl_2_, RT, 3 h.

As shown in Scheme 1, the complexes [Ag(ATV)(PPh_3_)_2_] (**1**), [Au(ATV)(PPh_3_)]·2H_2_O (**2**), and [Cu(ATV)(PPh_3_)_2_] (**3**) were synthesized using a 1:1 stochiometric ratio of ATV with the respective transition metal complex ([Ag(PPh_3_)_2_NO_3_], [Au(PPh_3_)Cl], [Cu(PPh_3_)_2_NO_3_]), in the presence of a strong base at room temperature. These complexes were obtained with good yields (46% to 85%). The use of a strong base, such as KOH or NaOCH_3_, allowed the deprotonation of the hydroxyl group in ATV to form the quinone ion and thus to promote bidentate coordination and the formation of a more stable coordination mode. This method takes advantage of a chelate effect and a subsequent reduction in the reaction time.

Complexes **1**, **2**, and **3** were air-stable, had enough solubility in common organic solvents (dichloromethane, chloroform, dimethyl sulfoxide, acetone), and were partially soluble in methanol, ethanol, and acetonitrile. The elemental analyses were found to lie within 0.4% of the calculated formulas for the proposed structures. Another analytical characterization of these complexes was their molar conductivity (ΛM), which for all three complexes was found to lie within the range of neutral compounds in DMSO^36^ (see Experimental Section). The chemical and physical characterization of ATV and the metal–ATV complexes are presented in the Supporting Information (Figures S1–S16). The UV–Vis spectra of the metal–ATV complexes presented only one strong absorption band each — at 521, 526, and 503 nm for complexes **1**, **2**, and **3**, respectively — which showed significant bathochromic shift in comparison to the metal-free ATV (λ = 400 nm). We attributed this band to a metal-to-ligand charge transfer (MLCT metal →π ATV naphthoquinone)^37^ (Figures S3, S26–S28).

The presence of the functional groups carbonyl and hydroxyl in the ATV meant infrared (IR) spectroscopy could provide significant information about the coordination mode of ATV to the metals. The IR bands corresponding to carbonyl (C_1_=O) and the deprotonation of the hydroxyl groups (C_2_–O) in ATV underwent significant changes upon metal coordination (Figures S5–S7). The distinctive OH band (3377 cm^–1^), which is easily recognized in free ATV (Figure S4), was absent in the three complexes. In contrast, a slight to significant increase in the intensity of the *v*(C_2_–O) and δ(C_2_–O) bands was observed for all three metal–ATV complexes in comparison to metal-free ATV (Table 1), indicating the coordination of the oxygen anion at the C_2_ position to the metal in the compounds. The values summarized in Table 1 further revealed a strong shift in the frequency values (*v*) of the carbonyl group (C_1_=O) to lower wavenumbers in complexes **1** and **3**: *v* = 133 cm^–1^ for **1** and *v* = 127 cm^–1^ for **3**, when compared to ATV. These results suggest that ATV was coordinated in a bidentate mode by the carbonyl group at position C_1_ (C_1_=O–M) and the oxygen atom at position C_2_ (M–O–C_2_). Moreover, much higher values of Δ*v* in the metal complexes (around 100–150 cm^–1^) suggest that the ATV was in a state of semiquinone oxidation (mono ion, in resonance between C_1_–O and C_2_–O).^38,39^ Regarding complex **2**, the bands corresponding to the carbonyl groups C_1_=O and C_4_=O showed only a slight change (Δ*v* = 7 cm^–1^) or no displacement at all (Δ*v* = 0 cm^–1^), while a significant displacement of the C_2_–O band (Δ*v* = 30 cm^–1^) was observed (Table 1, Figure S5**)**, indicating that in the gold(I) complex, ATV was coordinated as a monodentate ligand. To the best of our knowledge, monodentate coordination has not been previously reported for metal complexes containing this type of ligand.^1^H, ^13^C{^1^H}, and ^31^P{^1^H} NMR were further recorded to characterize the coordination modes of ATV to the transition metals in these diamagnetic complexes. According to the analysis of the ^1^H NMR spectra of the metal–ATV complexes (**1**–**3**), ATV was coordinated to the metals, since all the protons shifted to higher-field, particularly the H_5_–H_8_ protons in the naphthoquinone ring (between 0.01 and 0.32 ppm, Table 1). A greater shift in the displacement of H_8_ could be observed when ATV was coordinated as a bidentate ligand, such as in complex **1**, with a Δδ value of 0.30 ppm, or in **3** (where ATV was coordinated as a bidentate ligand), with a Δδ value of 0.29 ppm. In contrast, in compound **2** (where ATV was coordinated as a monodentate ligand), a much lower Δδ value of 0.01 ppm was observed. Calculations of ^1^H NMR peak integration confirmed a 2:1 ratio of triphenylphosphine to ATV for complexes **1** and **3**, but a 1:1 ratio for the gold complex (**2**) (Figures S8, S11, and S14).

**Table 1:** Characterization of Metal–ATV Complexes **1**–**3**

selected absorption bands (cm^–1^)						selected chemical shifts [δ(ppm) in DMSO-*d*_6_ ]						
IR						^1^H NMR				^13^C NMR		
compd.	OH	C_1_=O	C_4_=O	δC_2_–O)	*v*(C_2_–O)	H_5_	H_8_	H_6_	H_7_	C_4_=O	C_1_=O	C_2_–O
ATV	3377	1660	1647	1369	1278	7.99	7.97	7.83	7.77	184.4	181.3	155.4
**1**^a^	NA	1522	1652, 1645	1360	1281	7.84	7.67	7.62	7.44	186.7	178.7	159.5
**2**^a^	NA	1653	1647	1339	1265	7.98	7.98	7.83	7.76	184.1	182.3	156.9
**3**^a^	NA	1533	1653, 1647	1384	1284	7.85	7.68	7.62	7.43	186.7	178.7	159.5

a ATV compounds, atovaquone: **1**, [Ag(ATV)(PPh_3_)_2_]; **2**, [Au(ATV)(PPh_3_)]; **3**, [Cu(ATV)(PPh_3_)_2_]. NA: Does not appear. IR: the solid samples were measured in an FT–IR spectrometer with attenuated total reflectance accessory.

Examination of the ^13^C{^1^H}-NMR spectra for the compounds (Figures S9, S12, S15) showed that the signals corresponding to the carbons of the C_1_=O and C_2_–O functional groups exhibited the largest shifts compared to metal-free ATV (Table 1). For the silver and copper complexes (**1** and **3**), the C_2_–O signals displayed more lower-field shifts (Δδ of 4.1 ppm) than the gold complex (Δδ= 1.5 ppm) (Figures S9, S12, S15), indicating that the C2 carbon is more deshielded after the coordination of ATV to the metal center. Regarding the shift of the peak corresponding to the carbonyl group C_1_=O, a higher field shift (Δδ = 2.6 ppm) was observed in the silver and copper complexes. While the gold complex showed a downfield chemical shift variation (Δδ = 1.0 ppm) compared to free ATV. These data also confirm that ATV was coordinated as a bidentate ligand to Ag(I) and Cu(I) and as a monodentate ligand to Au(I). In the case of the C_4_=O carbon, its chemical shift variations are in the opposite direction to those observed for the C_1_=O group.

The ^31^P{^1^H}-NMR spectra of all three complexes confirmed the presence of the phosphine ligands coordinated to the metal ion (Figures S10, S13, S16). A characteristic singlet was observed at 32.66 ppm for **1**, 9.13 ppm for **2**, and −1.70 ppm for **3**, which were shifted from the precursor M–PPh_3_ complexes. This same profile of displacement has been reported for metal–phosphine complexes in the literature.^30,34,40^ In the case of complex **3**, a broad signal was further observed due to the quadrupolar interactions generated by the metal center, where both nuclei (^63^Cu and ^65^Cu) display a spin value of 3/2.^41^ No peak corresponding to triphenylphosphine oxide was observed in any spectrum of these complexes (**1**–**3**), which also indicates their purity.

### Single-Crystal X-ray Diffraction

Single-crystal X-ray diffraction was employed to characterize the crystal structure of complexes Ag(I), Au(I), and Cu(I), labeled **1**, **2**, and **3**, respectively. Crystallographic data for these metal–ATV complexes showed three different crystal systems (orthorhombic, monoclinic, and triclinic). As far as we currently know (CSD version 5.43, updated in 2022), these are the first crystal structures containing ATV and these metals reported in the crystallography database. Table S2 contains a summary of their structural refinement and crystallographic data.

**Figure 1:**
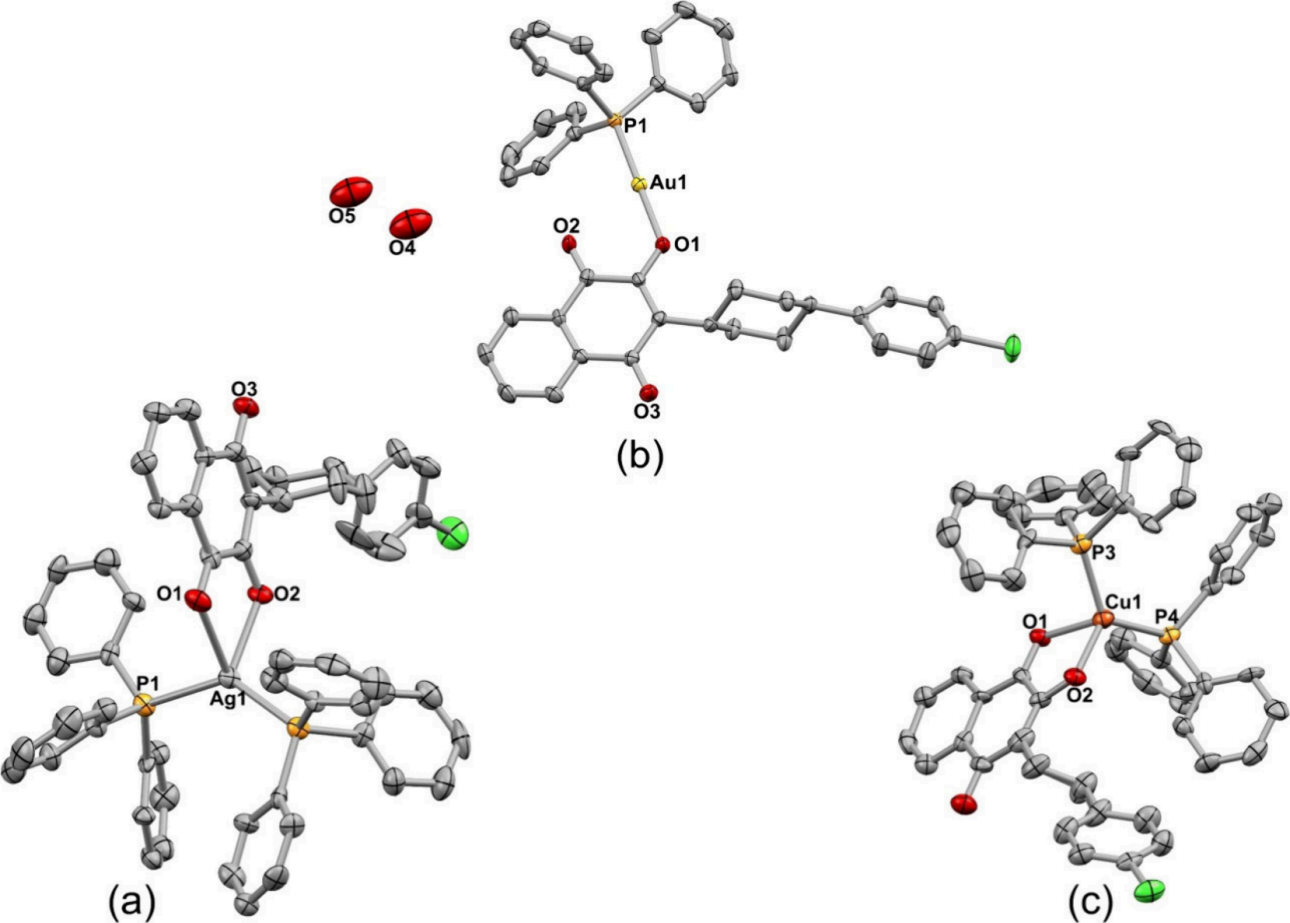
Crystal structure of complexes **1**, **2**, and **3**: (a) [Ag(ATV)(PPh_3_)_2_] (**1**), (b) [Au(ATV)(PPh_3_)]·2H_2_O (**2**), and (c) [Cu(ATV)(PPh_3_)_2_] (**3**). All of the thermal ellipsoids were drawn with 25% probability, and H atoms were omitted for clarity.

Table S3 shows selected angles and distances in the metal coordination environment. Complex **1** crystallized in the monoclinic system and the noncentrosymmetric space group P21, where the asymmetry unit consists of two crystallographically independent units [Ag(ATV)(PPh_3_)_2_]. Panel A of Figure 1 shows a fragment of this crystal structure (for further inspection, see Figures S17–S19). Each Ag^+^ ion is coordinated to two PPh_3_ molecules and one ATV ligand in the crystal structure with the following bond lengths: Ag1–O1 (2.558(17) Å) and Ag1–O2 (2.303(16) Å) from two oxygen atoms, as well as Ag1–P1 (2.444(6) Å) and Ag1–P2 (2.434(6) Å) from two phosphorus atoms. Therefore, Ag(I) is a 4-coordinated structure with distorted tetrahedral coordination geometry. The second [Ag(ATV)(PPh_3_)_2_] unit also showed a 4-coordinated structure and similar parameters for two PPh_3_ and one ATV ligand (Table S3; Figure S17). Overall, the geometry and coordination parameters of ATV are consistent with those found in other structurally related compounds.^42−44^ The neutrality of this compound is achieved by the loss of a hydrogen atom in the – OH group from the ATV ligand, as suggested by the similar distances between the oxygen atoms derived from the hydroxynaphthoquinone (O1–C75, O2–C74, O4–C37, and O5–C36 with values at 1.22(3), 1.27(3), 1.27(3), and 1.27(3) Å, respectively).

The crystal structure of complex **2** is also shown in panel B of Figure 1. The charge-neutral asymmetric unit is composed of an [Au(ATV)(PPh3)] unit. Gold is expected to have a + 1 oxidation state, with ATV acting as an anionic molecule and O1 and P1 atoms forming a 179.54(19)° angle with the Au(I) ion (linear coordination geometry), along with disordered solvent molecules in the structure. The O4 and O5 atoms are disordered water molecules in the lattice. To confirm the oxidation state of Au based on structural parameters, a three-dimensional geometrical analysis was employed using Voronoi–Dirichlet partition (VDP) of the crystal space.^45^ By assuming that variation of the periods and groups of the Periodic Table resemble the trends of atomic and ionic radii versus the fluctuation in the values of the radii of spherical domains (Rsd), it is expected that the lower the Rsd values, the greater the oxidation state of a given element. For complex **2**, the total VDP volume equaled 18.35 Å^3^, with Rsd of 1.6363 Å (calculated using ToposPro software^46^). When compared to the values computed for the gold crystal structure in oxidation state II (very rare labile species in a nitrogen environment; VDP = 15.00 Å^3^;, Rsd = 1.5300 Å)^47^ and III (VDP = 11.86–14.15 Å^3^;, Rsd = 1.4148–1.5003 Å),^48,49^ these data indicate that in fact the Rsd and VDP were smaller, strengthening our proposal regarding the oxidation state of Au in complex **2**.

Moreover, the application of a 2-fold rotation axis leads to the formation of a symmetric neighboring [Au(ATV)(PPh_3_)] unit (Figure S20). The Au–Au contact distance of 3.2547 (7) Å indicates the presence of aurophilic interactions, notable for being shorter than the sum of the van der Waals radii of the two gold atoms involved^50^ (Table S3). Like the Ag–ATV, complex 3 has a [Cu(ATV)_2_(PPh_3_)_2_] unit (Figure 1, panel C), where the Cu ion is coordinated to two PPh_3_ and one ATV, suggesting tetrahedral geometry (Figure 1, Table S3). Similar to other compounds, ATV is deprotonated in the crystal structure, and the structural parameters of the PPh_3_ ligand in all the compounds are comparable to the structures reported previously in the literature.^51^

#### Chemical Stability in Solution

Three distinct methodologies were used to investigate the in-solution stability of metal–ATV complexes **1**–**3**, namely ultraviolet–visible (UV–vis) spectra, NMR, and molar conductivity. All experiments were performed in dimethyl sulfoxide (DMSO) and monitored for at least 72 h, a time frame which coincides with antimalarial drug treatment.

The molar conductivity values did not differ significantly up to 15 days (Table S2). Furthermore, no significant changes in the ^1^H NMR and ^31^P{^1^H} NMR spectra of the complexes were observed up to 72 h in comparison to freshly dissolved solution, denoted as time 0 h (Figures S20–S25). However, for the ^31^P{^1^H} NMR spectrum of the complex **3**, it is observed an increasing intensity of the singlet at 28 ppm that can be attributed to oxidized PPh_3_, which may be leaving the coordination sphere, but this is relative low when compared with the signal of the complex at −1.76 ppm. Both molar conductivity and NMR data therefore indicate that the metal complexes remained intact in this condition. The observed reactivity in DMSO is likely related to the fact that the metal atoms are bonded to phosphine ligands, hindering any oxidation of the metal center.

As for the UV–vis analyses, these were used to assess the stability of metal–ATV complexes **1**–**3** in a solution of pure DMSO or DMSO mixed with aqueous media. To this end, three different conditions were employed in parallel experiments. In pure DMSO, modifications were observed in the electronic spectra for complexes **2** and **3**, primarily within the interval of 0–24 h (Figures S26–S28). The same behavior was observed under the condition of 90% DMSO mixed with 10% water, with a major change in the spectra seen for complex **3** (Figures S29–S31). The small change in absorbance observed can be attributed to dynamic equilibrium processes or structural reorganization of the metal complexes. These processes may include partial dissociation of ligands, reorientation of solvent molecules around the metal ion, or even conformational changes of the metal complexes. In the third condition, 90% DMSO mixed with 10% RPMI culture medium, minor modifications were observed in the spectra for both systems, the metal-free ATV and the metal–ATV complexes (Figures S32–S35), indicating that under these conditions, the compounds remain intact for up to 72 h. Highlighting that these conditions are the most similar to those employed in the determination of biological activity. Based on this, we concluded that in mixed aqueous/organic media, ATV and complexes **1**–**3** may undergo slight structural modifications, but no significant ligand exchange reactions occur, as previously observed in the stability studies by NMR.

#### Drug Interaction with FePPIX and Inhibition of β-Hematin Crystals

**Figure 2:**
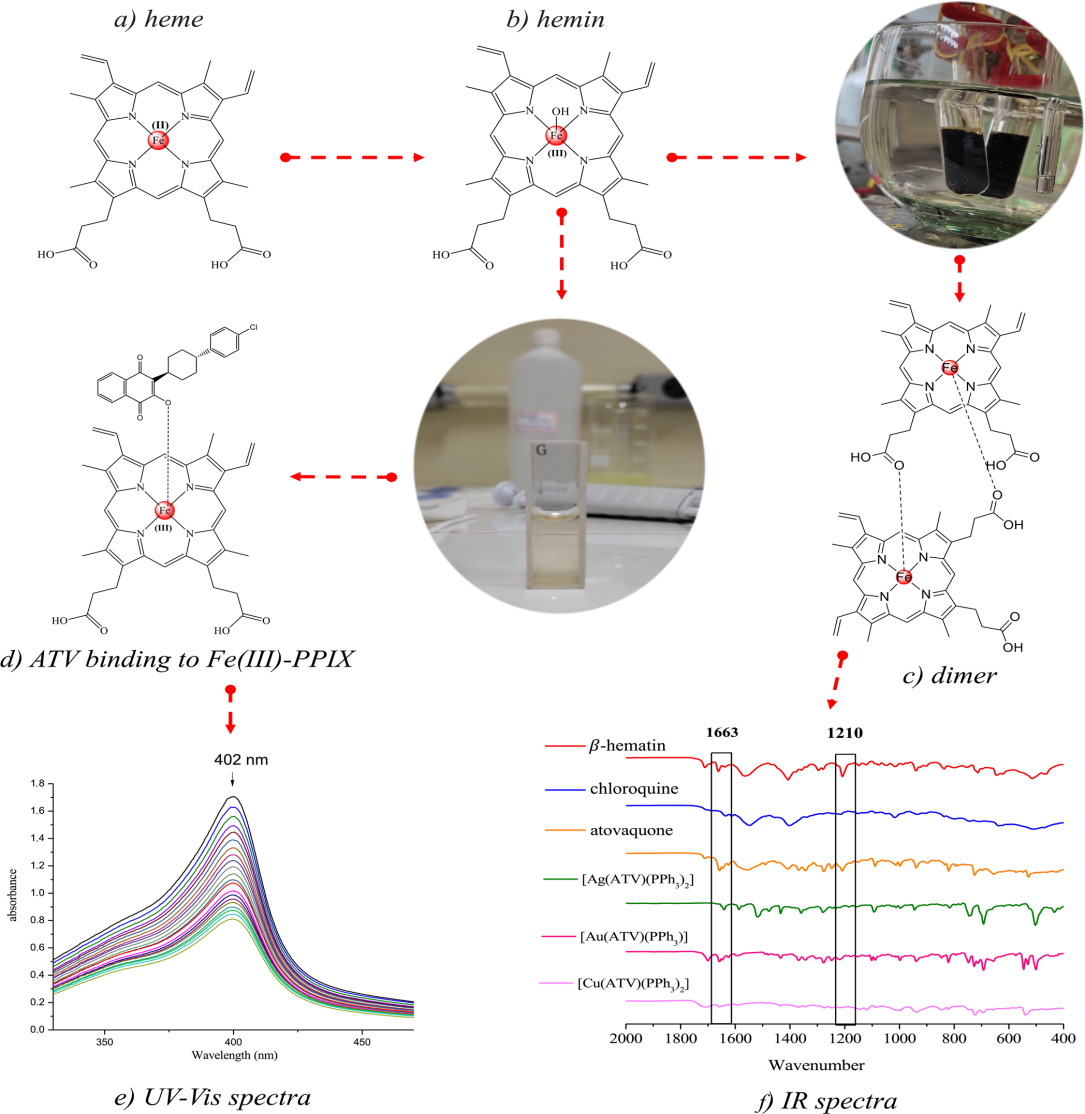
Chemical structures of (a) heme, (b) hemin, and (c) hematin dimers (β-hematin); (d) generic coordination of FePPIX to ATV (putative structure); (e) spectroscopic titration of Soret band at 402 nm to calculate association constants (log *K*); (f) representative IR spectra of β-hematin formation in acidic-buffer pH in the absence or presence of compounds at a 1:3 hematin/compound ratio.

Heme detoxification is a pivotal process for the growth of asexual blood stages of *Plasmodium*, and its inhibition is one of the main mechanisms of action for 4-aminoquinoline drugs such as chloroquine and amodiaquine. We examined whether ATV and its metal complexes **1**–**3** could suppress the mechanism of heme detoxification into hemozoin crystals. To assess this, we evaluated the ability of ATV and complexes **1**–**3** to bind with soluble hemin and further inhibit its polymerization into hemozoin crystals as a proxy model of the heme detoxification that takes place in the parasite cell (Figure 2a–c).^52^^53^^54^^55^

First, the binding of compounds to hemin [Fe(III)PPIX, ferriprotoporphyrin IX] was studied by spectroscopic titration of the Soret band determined at 402 nm. The association constants (Log *K*) of the compounds with hemin were determined at pH 7.4 using a 1:1 complexation model with the equation proposed by Egan et al.^52^ for the nonlinear least-squares fitting (Figure 2, Table 2). Figure 2e shows the titration of hemin with ATV. The hypochromic effect on the Soret band of hemin caused by ATV was 42% until saturation point was reached; the same behavior was also observed for complexes **1**–**3**, yielding hypochromic effects of 37%, 51%, and 38%, respectively (Figures S36–S38).

**Table 2:** Cytotoxicity in Mammalian Cells, *in Vitro* Antiparasitic Activity against Asexual Blood Stages of *P. falciparum*, Selectivity Index, and Interaction with Hemin

	mammalian cells CC_50_ in μM (±SEM)^a^		*P. falciparum* IC_50_ in nM (±SEM)^b^			log *D*		
compounds	J774	HepG2	3D7	W2	S.I.^c^	pH 5	pH 7	log *K*^d^
ATV	19.8 ± 1.6	32.4 ± 4.8	2.4 ± 1.2	2.1 ± 0.9	8250	–1.17	–1.15	3.64 ± 0.10
CQ	50.5 ± 8.9	∼80	23.8 ± 5.5	526 ± 126	2121	–1.25	0.98	4.78 ± 0.01
**1**	9.5 ± 2.2	14.6 ± 0.9	30.5 ± 9.7	26.5 ± 7.8	311	–2.12	0.08	4.33 ± 0.05
**2**	10.8 ± 3.4	8.9 ± 2.7	8.1 ± 2.7	5.2 ± 2.2	1333	–1.87	–1.71	4.46 ± 0.01
**3**	36.1 ± 3.9	44.3 ± 7.7	30.7 ± 10.7	26.3 ± 6.9	1175	–5.18	1.06	3.97 ± 0.01
AuClPPh_3_	4.1 ± 0.8	ND	1431 ± 309	3294 ± 629	2.8	ND	ND	ND
[Cu(PPh_3_)_2_NO_3_]	>80	ND	>20000	>20000	ND	ND	ND	ND
DOX	0.44 ± 0.31	<0.12	ND	ND	ND	ND	ND	ND

a Values are the half-maximal cytotoxicity concentration (CC_50_) and are expressed as the means of three experiments (each concentration in triplicate).

b Values are the half-maximal inhibitory concentration (IC_50_) and are expressed as the means of five to seven experiments (each concentration in duplicate).

c Selectivity index (S.I.) was determined as CC_50_ (J774)/IC_50_(3D7).

d Association constant (log *K*) values of drug binding to hemin are the mean and SEM of three independent experiments. [a,b] Determined after 72 h of drug incubation. J774, murine macrophage line; HepG2, human hepatocellular carcinoma line. 3D7 = CQ-susceptible *P. falciparum* strain; W2 = CQ-resistant *P. falciparum* strain. **1**, [Ag(ATV)(PPh_3_)_2_]; **2**, [Au(ATV)(PPh_3_)]; **3**, [Cu(ATV)(PPh_3_)_2_]; SEM = standard errors of the means; ATV = atovaquone; CQ = chloroquine; Dox = Doxorubicin; N.D. = not determined. *K* is the association constant between hemin chloride and the compounds.

Next, we inspected the Log *K* values of the compounds and compared them with the reference drug chloroquine. Metal complexes **1**–**3** interacted with hemin in a similar way to metal-free ATV, regardless of the transition metal. The range of Log *K* values was comparable to that obtained for chloroquine. Complexes [Ag(ATV)(PPh_3_)_2_](**1**) and [Au(ATV)(PPh_3_)] (**2**), exhibited Log *K* values comparable to those found for chloroquine and ATV, whereas [Cu(ATV)(PPh_3_)_2_] (**3**) showed a slightly lower Log *K* value.

The ability of these compounds to inhibit the formation of β-hematin crystals was investigated by IR spectroscopy (Figure 2f). Of note, β-hematin crystal formation was assayed in an acid-buffered medium, while hemin titration was assayed at neutral pH. In the absence of drugs, the IR spectrum of β-hematin crystals typically displays two characteristic absorption bands of the dimer: one at 1663 cm^–1^, relating to stretching (C = O), and another at 1210 cm^–1^, attributed to the stretching (C–O) of the coordinated carboxylate group to the Fe(III). In our assays, in comparison to drug-free conditions, the spectra of β-hematin formation in the presence of chloroquine decreased simultaneously in these two bands. For both ATV and complexes **1**–**3**, there may have been overlapping between a decrease in the (C = O) band of heme species and a (C = O) band of quinone. However, there was no danger of overlaps in the (C–O) band of heme species at 1210 cm^–1^, since these species are absent from the drugs. By monitoring this specific band, we inferred those complexes **1–3** were able to inhibit β-hematin crystal formation, but ATV were unable to inhibit β-hematin crystal formation at this hematin-to-drug ratio.

### Antimalarial Activity, Cytotoxicity to Mammalian Cells, and Selectivity Index

We determined the half-maximal inhibitory concentration (IC_50_) of the complexes against asexual blood stages of P. falciparum using CQ-susceptible and CQ-resistant strains (Table 2). By comparing the IC_50_ values for the different strains, we inferred that all the metal complexes were equally potent in inhibiting drug-susceptible and drug-resistant strains, indicating that the conjugation of ATV into metal complexes does not cause cross-resistance with CQ.^56^ The potency of the metal complexes to inhibit parasite growth was also examined in comparison with ATV. We observed that the complexes were equipotent or slightly less potent than ATV. Complex **2**, which was equipotent, had the ATV coordinated in monodentate mode to the metal (Au-ATV). The metal–ATV complexes in which ATV was coordinated in bidentate mode (Ag–ATV [**1**] and Cu–ATV [**3**]) were less potent than ATV.

Having determined the antiplasmodial activity, we aimed to understand whether the conjugation of ATV into metal complexes alters its selectivity index. To this end, cytotoxicity in mammalian cells was determined by calculating the drug concentration required to achieve 50% cytotoxicity (CC_50_). Cell lines J774 and HepG2 were incubated with the drug for 72 h, the same time frame as employed to determine antimalarial activity. Cytotoxicity was evaluated in parallel with ATV as a representative parental drug, while doxorubicin was used as a standard cytotoxic drug. These experiments were also used to estimate the *in vitro* selectivity index (Table 2). In general, ATV and its complexes were less cytotoxic than doxorubicin. In contrast, the complexes displayed twice or three times the cytotoxicity of ATV for mammalian cells. The complexes were also found to be less cytotoxic than the ATV-free gold precursor AuClPPh_3_, which appears to be a highly reactive and cytotoxic agent.^29^ By determining the selectivity indices, we confirmed that the metal complexes had a selective effect in inhibiting parasite growth rather than being cytotoxic for mammalian cells — a profile similarly observed for ATV.

### Distribution Coefficient (log *D*)

The distribution coefficient (Log D), a proxy parameter to estimate drug lipophilicity, was assessed for ATV and its metal complexes **1**–**3**. Log D was determined at both pH 5 and 7 and the values are presented in Table 2. At pH 5, all the complexes exhibited negative values within a similar range to those observed for chloroquine. At pH 7, only ATV and the gold complex (**2**) displayed negative Log D values. This suggests that ATV is a relatively poor lipophilic drug and that its coordination resulting in complex **2** does not increase its lipophilicity. In contrast, complex **1** and, to a lesser extent, complex **3** presented positive Log D values in a range similar to those of chloroquine, suggesting these drugs are more lipophilic.

### Structure–Activity Relationships

Typically, a structure–activity relationship can be discussed on the basis of the geometry of the metal complex, the redox state of the metal, and the composition and/or position of the coligands.^56,57^ Complex **2** has a linear geometry, with ATV coordinated in a monodentate mode. This complex displayed equal potency to ATV in inhibiting parasite growth. In contrast, the silver(I) and copper(I) complexes have distorted tetrahedral coordination geometry, and both were less potent than ATV. Given that the redox state of all the metals is the same (I) and the complexes have identical ligands (ATV and phosphine), added to the fact that the ATV-free metallic precursors are devoid of significant antiplasmodial activity, our interpretation is that the monodentate complex was more potent than the bidentate complexes because of the difference in the way ATV dissociates from the coordination compounds in these different types of complex.

**Figure 3:**
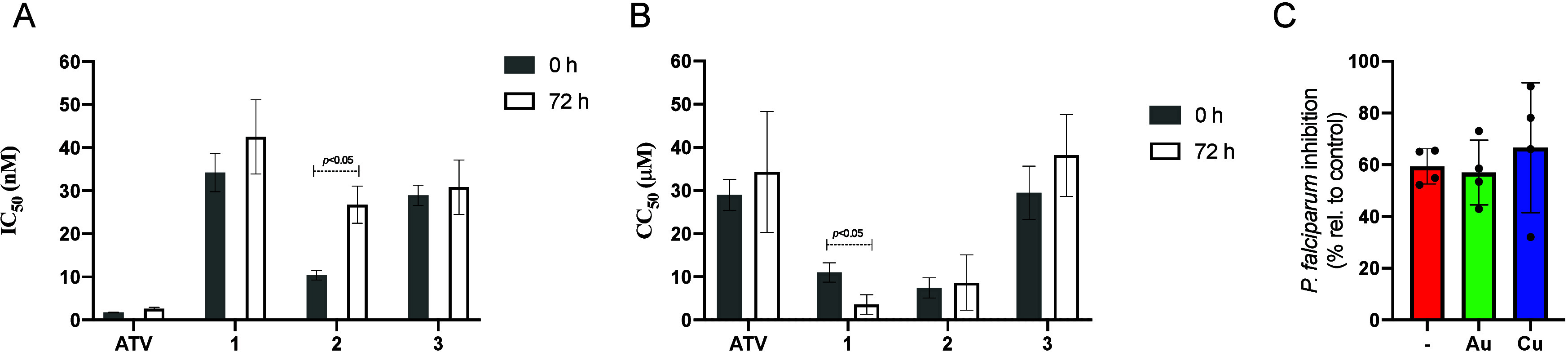
(A) Antiplasmodial activity of the compounds against the 3D7 strain of *P. falciparum*. (B) Cytotoxicity against the J774 cell line. In both cases, the compounds were diluted in cell culture and incubated for 0 or 72 h before use in the assays. Panel C shows the percentage of parasite growth inhibition against the 3D7 strain of *P. falciparum* of 1.0 nM ATV in the presence or absence of metal precursors of Au [Au(PPh_3_)Cl] and Cu [Cu(PPh_3_)_2_NO_3_] added at 1000 nM. In panels A and C, parasite viability was measured by HRP2 ELISA-based assay, and IC_50_ values were calculated. In panel B, cell viability was measured by CellTiter-Glo, and IC_50_ values were calculated. In panels A and B, the values are the mean and standard deviation of two independent experiments, using each concentration of compounds in duplicate. In panel C, the values are the mean and standard deviation of one experiment, using each concentration of compounds in quadruplicate. *p < 0.05; indicating significant difference by Student’s *t* test.

To understand the reason for the difference in antiplasmodial potency between monodentate complex **2** and bidentate complexes **1** and **3**, we incubated all three metal complexes in cell culture media for different times before assessing their antiplasmodial activity (Figure 3A). The percentage of parasite growth inhibition in samples of cell culture medium containing complex **2** was reduced after 72 h incubation in comparison to freshly prepared samples (0 h). In contrast, parasite growth inhibition remained unchanged for complexes **1** and **3** at 0 and 72 h. We interpret the loss in antiplasmodial activity for complex **2** as potentially resulting from ligand exchange reactions occurring in the culture medium before drug internalization into parasite cells. This denotes a higher lability for the monodentate complex **2** than for the bidentate complexes **1** and **3**.

To further shed light on this, we performed the same experiment described above but assessing cytotoxicity in J744 cells (Figure 3B). The cell viability of samples of cell culture medium containing complex **1** was affected after 72 h incubation in comparison to freshly prepared samples (0 h), namely, complex **1** became less cytotoxic under preincubation. In contrast, cell viability remained unchanged for complexes **2** and **3** between 0 and 72 h. We suggest the increased cytotoxicity for complex **1** could be caused by the formation of metallic species more reactive and labile for ligand exchange reactions. As for the bidentate complexes **1** and **3**, overall they demonstrated greater stability in cell culture settings than the monodentate complex **2**. Our data also confirm not only drug stability but also the reactivity of the resulting products as important parameters.

After ascertaining the existence of an association between the reactivity of the metal complexes and their pharmacological activity, we decided to evaluate whether they could be generated *in situ* upon coincubation of ATV and the respective metal precursors in the cell culture medium. In this set of experiments, ATV was added at 1.0 nM, while the metal precursors of gold(I) [Au(PPh_3_)Cl] and copper(I) [Cu(PPh_3_)_2_NO_3_] were added at 1000 nM (Figure 3C). We did not observe a difference in the parasite growth inhibition of ATV incubated in the absence or presence of the metal precursors. Our interpretation of this result is that the profile of antiplasmodial activity for the metal complexes is based on their structures rather than the contribution of each component, namely, ATV and the metal ion.

## Conclusions

Three metal–ATV complexes containing triphenylphosphine were synthesized and characterized in solid state and in solution. Their structures were elucidated by analytical and spectroscopy techniques and their coordination spheres were further elucidated by single-crystal X-ray diffraction. This revealed the coordination mode of ATV as a bidentate ligand to copper(I) and silver(I) ions and a monodentate ligand to gold(I) ions. Complexes **1**–**3** remained stable in both solid state and solution. ATV and complexes **1**–**3** can bind to hemin, denoting the affinity of this class of compounds toward heme species. All complexes **1–3** can inhibit the formation of β-hematin crystals. The complexes showed potent antiplasmodial activity against CQ-sensitive and CQ-resistant strains of *P. falciparum* in the nanomolar range; no cross-resistance with CQ was inferred. Complex **2** showed the highest potency, despite being less potent than ATV. Our chemical design therefore consists of a first step in elucidating the synthesis, characterization, and activity of antimalarial quinones as a potential and emerging class of drugs against malaria.

## Experimental Section

Solvents (methanol, ethanol, dichloromethane, chloroform, acetonitrile, dimethyl sulfoxide) were used without purification. The three complexes ([Ag(PPh_3_)_2_NO_3_], [Au(PPh_3_)Cl], and [Cu(PPh_3_)_2_NO_3_]) were synthesized following reported procedures.^58−62^ Atovaquone (ATV) was used as received from Sigma-Aldrich. ^1^H, ^13^C{^1^H}, and ^31^P{^1^H} nuclear magnetic resonance spectra were obtained in a Bruker Advance III HD 500 spectrometer using DMSO-*d*_6_ as a solvent. Chemical displacement was reported as δ ppm (integration, multiplicity, attribution). Fourier-transform infrared spectroscopy (FTIR) was recorded in solid state in the 400–4000 cm^–1^ region with an average of 128 scans by using a Bruker Alpha FT-IR spectrometer with an attenuated total reflectance accessory with 4 cm^–1^ resolution. The UV–visible absorption spectra were obtained using a UV-1800 Shimadzu spectrometer with 1 nm spectral bandwidth in the 200–1100 nm range. Elemental analyses of carbon, hydrogen, and nitrogen were conducted using a Thermo Scientific CHNS-O Flash 2000 analyzer. Conductivities values were measured in a MS Tecnopon NI-CVM instrument on DMSO solutions of 1 mM.

### X-ray Crystallography

Single crystals of complexes were selected for X-ray diffraction studies and the data were collected in a SuperNova, Dual AtlasS2 with Mo*Kα* radiation (λ = 0.7107 Å) for complex **2** and Cu*Kα* (λ = 1.5406 Å) for complexes **1** and **3**, at a 291.4(2) K setting. Data reduction was performed using crysAlisPro software,^62^ which corrects Lorentz polarization and absorption effects. Using Olex2,^63^ the crystal structures were solved with the Superflip^64^ solution program, using the Charge Flipping solution method. The model was refined with the 2014/7 version of ShelXL^65^ using full-matrix least-squares minimization on *F*^2^. All non-hydrogen atoms were refined with anisotropic thermal parameters. The absolute structure was arbitrarily determined. (For more details of the data crystal, see the Supporting Information). Crystallographic data for all the compounds were deposited at The Cambridge Crystallographic Data Center (www.ccdc.cam.ac.uk/data_request/cif) and can be obtained free of charge under deposition number 2307576–2307578.

#### *Synthesis of* [Ag(ATV)(PPh_3_)_2_] (**1**)

A solution of KOH (0.0076 g, 0.1363 mmol) in methanol (5 mL) was added dropwise to a solution of ATV (0.0500 g, 0.1363 mmol) in CH_2_Cl_2_ (10 mL) at room temperature under magnetic stirring. The color of the solution changed from yellow to red. After 1 h, a solution of [Ag(PPh_3)2_]NO_3_ (0.0946 g, 0.1363 mmol) in CH_2_Cl_2_ (5 mL) was slowly added and the reaction was kept under stirring for another 3 h. The resulting dark purple solution was left at room temperature, and after several days crystals were formed and filtered. Yield: 85% (0.0621g). Elemental analyses (%) calc. for C_58_H_48_O_3_P_2_ClAg: C 69.78; H 4.85. Found: C 69.70; H 4.74. Molar conductivity in DMSO: Λ_M_ = 12.81. UV–vis (DMSO) ([assignment; ε, _._M^–1^cm^–1^] (λ_max_ 521 nm (π → π*, 3819.9)). IR (cm^–1^): *v*(C–H) 3049; *v*(C–H) 2926; *v*(C–H) 2852; *v*(C = O) 1645; *v*(C = C) 1587, 1558; *v*(C = O) 1522; *ô*(C–H2) 1474, 1456, 1435; *ô*(OH) 1360; *v*(C–O) 1281 (s,b). ^1^H NMR (DMSO-*d*_6_) δ ppm (integral, multiplicity, attribution): 7.84 (1H, dd, H_5_); 7.67 (1H, dd, H_8_); 7.62 (1H, td, H_6_); 7.49 (6H, tb, H_PPh3_); 7.44 (1H, td, H_7_); 7.41–7.33 (24H, m, H_PPh3_); 7.32 (2H, dt, H_19,21_); 7.28 (2H, dt, H_18,22_); 3.12 (1H, tt, H_14_); 2.43 (2H, qd_,_H_13,15_); 1.81 (2H, d, H_13,15_); 1.46 (2H, d, H_12,16_); 1.44 (2H, m,H_12,16_). ^13^C{H}NMR (DMSO-*d*_6_) δ ppm (attribution): 186.72 (C_4_=O); 178.71 (C_1_= O); 159.50 (C_2_–O); 133.24 (Ar–C_6_H); 133.56 (6x o-PPh_3_Ar–CH); 133.43 (6x o-PPh_3_Ar–CH); 136.04 (Ar–C_7_H); 132.13 (3x Pp-PPh_3_Ar–CH); 131.94 (3x Pp-PPh_3_Ar–CH); 131.32 (Ar–C_20_);130.59 (6x p-PPh_3_Ar–CH); 130.10 (Ar–C_10_); 129.65 (Ar–C_9_) ; 129.13 (6*m*-PPh_3_Ar–CH); 129.05 (6x m-PPh_3_Ar–CH); 128.65 (2x Ar–C_19,21_H); 128.16 (2x Ar–C_18,22_H); 124.97 (C_3_); 124.37 (Ar–C_5_H) ; 122.31 (Ar- C_8_H); 132.13 (3x Pp-PPh_3_Ar −CH); 131.94 (3x Pp-PPh_3_Ar _–_ CH); 130.59 (6x p-PPh_3_Ar −CH); 129.13 (6x m-PPh_3_Ar–CH); 129.05 (6x m-PPh_3_Ar–CH); 124.37 (Ar–C_5_H); 122.31 (Ar–C_8_H) . ^31^P{^1^H} NMR (DMSO-*d*_6_), δ ppm (multiplicity, attribution): 9.13 (s. PPh_3_). The structure of the compound was determined by X-ray diffraction.

#### Synthesis of [Au(ATV)(PPh_3_)]·2H_2_O (**2**)

Powder AgNO_3_ (0.0231 g, 0.1359 mmol) was added to a solution of [Au(PPh_3_)Cl] (0.0674 g, 0.1362 mmol) in dichloromethane (20 mL) and was stirred at room temperature for 24 h. The solution was filtered off and the solid was added to a solution containing ATV (0.0500 g, 0.1363 mmol) previously treated with sodium methoxide (0.0073 g, 0.1351 mmol) in dichloromethane (10 mL). The color of the reaction immediately changed from dark red to reddish orange. The reaction was left at room temperature under stirring for 3 h. It was then filtered, and the solution was left at room temperature, forming crystals after several days. Yield: 87% (0.098 g). Elemental analyses (%) calc. for C_40_H_37_O_5_ClPAu: C 55.79, H 4.33. Found: C 55.99, H4.14. Molar conductivity in DMSO: Λ_M_ = 12.69. UV–vis (DMSO) ([assignment; ε, M^–1^ cm^–1^] (λ_max_ 525,5 nm (π → π*, 443.95)). IR (cm^–1^): *v* (C–H) 2915; *v* (C–H) 2851; *v*(C = O) 1653; *v*(C = O) 1647; *v*(C = C) 1576, 1569; *ô*(CH_2_) 1489, 1473, 1456; *ô*(OH) 1362; *v*(C–O) 1265. ^1^H NMR (DMSO-*d*_6_) δ ppm (integral, multiplicity, attribution): 7.98 (2H, td, H_5_, H_8_); 7.83 (1H, td, H_6_); 7.76 (1H, td, H_7_); 7.70–7.49(11H, m, PPh_3_); 7.32 (4H, d, H_19,21_, H_18,22_); 3.08 (1H, tt, H_14_); 2.57 (1H, tt, H_11_); 2.26 (2H, qd, H_13,15_); 1.85 (2H, d, H_13,15_); 1.57 (2H, d, H_12,16_); 1.49 (2H, qd, H_12,16_). ^13^C{H}NMR (DMSO-*d*_6_) δ ppm (attribution): 184.10 (C_4_=O); 182.30 (C_1_=O); 156.93 (C_2_–O); 146.44 (Ar–C_17_); 134.40 (Ar–C_6_H); 133.87 (Ar–CH-PPh_3_); 133.76 (Ar–CH-PPh_3_); 132.71 (Ar–CH-PPh_3_); 132,63 (Ar–CH-PPh_3_); 132,33 (Ar–CH-PPh_3_); 130,27 (Ar–C_7_H) ; 129,95 (Ar–C_20_); 129.72 (Ar–CH-PPh_3_); 129.63 (Ar–CH-PPh_3_); 128.65 (2x Ar–C_19,21_H); 128.46 (Ar–C_10_) ; 128.16 (2x Ar–C_18,22_H); 127.97 (Ar–C_9_); 126.12 (C_3_); 125.81 (Ar–C_5_H) ; 125.35 (Ar–C_8_H) ; 42.72 (Alif-C_14_H) ; 34.11 (2 x Aliph- C_13_,15H_2_); 33.91 (Alif -C_11_H); 28.86 (2x Aliph- C_12_,16H_2_). ^31^P{^1^H} NMR (DMSO-*d*_6_), δ ppm (multiplicity, attribution): 32.66 (s. PPh_3_) . The structure of the compound was determined by X-ray diffraction.

#### Synthesis of [Cu(ATV)(PPh_3_)_2_] (**3**)

A solution of KOH (0.0076 g, 0.1354 mmol) in methanol (10 mL) was added drop by drop to a methanolic solution (60 mL) of ATV (0.0500 g, 0.1363 mmol) at room temperature under magnetic stirring. The color of the solution changed from yellow to red. After 1 h, a solution of [Cu(PPh_3_)_2_NO_3_] (0.0443 g, 0.1733 mmol) was slowly added. The resulting dark purple solution was left to rest at room temperature, forming crystals after several days. Yield: 72% (0.046 g). Elemental analyses (%) calc. for C_58_H_48_O_3_ClP_2_Cu: C 73.03, H 5.07. Found: C 72.92, H 4.76. Molar conductivity in DMSO: Λ_M_ = 3.71. UV–vis (DMSO) ([attribution]; ε_, ._M^–1^cm^–1^] (λ_max_ 503.5 nm (π → π*,4316)). IR (cm^–1^): *v*(C–H) 3055; *v*(C–H) 2922; *v*(C–H) 2854; *v*(C = O) 1635; *v*(C = C) 1587; *v*(C = O) 1533; (C–H) 1479, 1438; (OH) 1384, 1361; *v*(C–O) 1284, 1249, 1233. ^1^H NMR (DMSO-*d*_6_) δ ppm (multiplicity, integral, attribution): 7.85 (1H, d, H_5_); 7.68 (1H, d, H_8_); 7.62 (1H, tdb, H_6_); 7.41 (6H, m, HPPh_3_); 7.43 (1H, td, H_7_); 7.43–7.34 (24H, m, HPPh_3_); 7.33 (4H, d, H_19,21,18,22_); 3.09 (1H, tt, H_14_); 2.38 (2H, qd, H_13,15_); 1.80 (2H, d, H_13,15_); 1.43 (2H, d, H_12,16_); 1.45 (2H, m, H_12,16_). ^13^C{H}NMR (DMSO-*d*_6_) δ ppm (attribution): 186.72 (C_4_=O); 178.71 (C_1_=O); 159.50 (C_2_–O); 133.24 (Ar–C_6_H)_;_ 133.56 (6x PPh_3_Ar - CH); 133.43 (6x PPh_3_Ar–CH); 136.04 (Ar–C_7_H); 132.13 (3x PPh_3_Ar–CH); 131.94 (3x PPh_3_Ar–CH); 130.59 (6x PPh_3_Ar–CH); 129.13 (6x PPh_3_Ar–CH); 129.05 (6x PPh_3_Ar–CH); 124.37 (Ar–C_5_H); 122.31 (Ar–C_8_H). ^31^P{^1^H} NMR (DMSO-*d*_6_) δ ppm (multiplicity, attribution): −1.70 (s. PPh_3_). The structure of the compound was determined by X-ray diffraction.

#### Chemical Stability in Solution

For the stability studies by NMR spectroscopy, fresh solutions of the complexes in DMSO-*d*_6_ with concentrations of 22–25 mM were used. In the studies by UV–vis spectroscopy, three experimental conditions were employed: a) only DMSO, b) 90% DMSO/10% H_2_O, and c) 90% DMSO/10% RPMI 1640 (Roswell Park Memorial Institute) medium, with concentrations of the complexes **1**-**3** in 0.28–0.35 μM, 0.25–0.28 μM and 0.32–0.38 μM, respectively. Fresh solutions of the complexes dissolved in DMSO were always used and subsequently diluted with water or RPMI medium.

#### Interaction with Ferriprotoporphyrin IX

Following a procedure described in the literature,^52^ the association constant (Log *K*) of the drugs with ferriprotoporphyrin IX was determined in 40% aqueous DMSO. A stock solution of hemin chloride was prepared by adding 3.5 mg hemin to 10 mL DMSO and adjusted to pH 7.5. A working solution of hemin in 40% DMSO was further prepared by mixing 140 μL hemin stock solution, 5 mL distilled water, 3.86 mL DMSO, and 1 mL 0.2 M Tris (tris(hydroxymethyl)aminomethane) buffer. Aliquots of compounds were added to this working hemin solution. Blank solutions containing only hemin or compounds were prepared to subtract the absorbance of the compounds. Absorbance at the Soret band (402 nm) was measured in the presence and absence of compound. Binding affinity was determined using the equation A = (A_0_ + A∞K[C])/(1 + K[C]) for a 1:1 complexation model using nonlinear least-squares fitting, where A_0_ is the absorbance of Fe(III)PPIX, A∞ is the absorbance of the compound–hemin adduct at saturation, and *K* is the association constant. Three independent experiments were performed.

### Determination of β-Hematin Formation

The conversion of hematin into β-hematin crystals was determined by FTIR spectroscopy as described previously.^52^ In a microtube, 20 mg hemin and three equivalents of the compounds were dissolved in 3 mL 0.1 M NaOH solution and stirred for 30 min at 60 °C. Then, 0.3 mL 0.1 M HCl and 1.7 mL sodium acetate buffer (10 M, pH 5) were added at the same temperature. Chloroquine was employed at this same drug-to-hemin ratio. After incubation for 120 min, the reaction mixture was cooled on ice for 10 min and then centrifuged and washed with water to remove salts. Resulting crystals were dried and IR spectra were obtained using ATR mode.^66−68^

### Determination of Antiplasmodial Activity

Two strains were selected for the tests: the CQ-susceptible 3D7 strain (isolated in West Africa; obtained from MR4, VA, USA), and the CQ-resistant W2 strain (isolated in Indochina; obtained from MR4, VA, USA). Both were maintained in culture in RPMI 1640 (Invitrogen, Paisley, UK), supplemented with 10% human serum (Abcys S.A. Paris, France), and buffered with 25 mM HEPES and 25 mM NaHCO_3_. Parasites were grown in A-positive human blood (Etablissement Français du Sang, Marseille, France) in controlled atmospheric conditions that consisted of 10% O_2_, 5% CO_2_, and 85% N_2_ at 37 °C with 95% humidity. The two strains were synchronized twice with sorbitol before use,^69^ and clonality was verified every 15 days through PCR genotyping of the polymorphic genetic markers *msp1* and *msp2* and microsatellite loci.^70,71^ Compounds were resuspended in DMSO and then diluted in RPMI-DMSO (99*v*/1v) to obtain final concentrations ranging from 0.25 to 20000 nM.

For in vitro isotopic microtests, 25 μL/well of antimalarial drug and 200 μL/well of parasitized red blood cell suspension (final parasitemia 0.5%, final hematocrit 1.5%) were distributed into 96-well plates. The plates were incubated for 72 h at 37 °C in 85% N_2_, 10% O_2_, 5% CO_2_. After freezing and then thawing, the hemolyzed cultures were homogenized by vortexing the plates. The drug susceptibility assay was performed using the HRP2 ELISA-based assay of the Malaria Ag Celisa kit (ref KM2159, Cellabs PTY LDT, Brookvale, Australia), as described previously.^72^ The concentration at which the drugs were able to inhibit 50% parasite growth (IC_50_) was calculated with the inhibitory sigmoid *E*_max_ model, with estimates of IC_50_ obtained by nonlinear regression using a standard function of the R software (ICEstimator version 1.2). IC_50_ values were validated only if the optical density (OD) ratio (OD at concentration 0/OD at concentration max) was greater than 1.6 and the confidence interval ratio (upper 95% confidence interval of the IC_50_ estimate/lower 95% confidence interval of the IC_50_ estimate) was less than 2.0. IC_50_ are expressed as means of at least 5 to 7 independent experiments.

### Cytotoxicity in Mammalian Cells

In 96-well plates, 100 μL HepG2 (in RPMI) or J774 cells (in DMEM) were seeded per well at 4.0 × 10^4^ cell/mL. After incubation at 37 °C in 5% CO_2_ for 24 h, 100 μL drug-containing medium was added per well and the plates were incubated for 72 h. Each drug was tested at seven different concentrations (1.25–80 μM), each one in triplicate. Doxorubicin chloridrate (Eurofarma, São Paulo, Brazil) was used as a positive control, while untreated cells were employed as negative controls. Bioluminescence readings were performed using a Filtermax F5Multi-Mode instrument (Molecular Devices, Sunnyvale, CA) using the CellTiter-Glo kit (Promega Corporation, Madison, USA). Three independent experiments were performed and CC_50_ values were calculated.

### Distribution Coefficient (log *D*)

Buffer–*n*-octanol distribution coefficients were determined using the shaking flask method.^73^ A UV–vis calibration curve was prepared in the 10–100 μM range in *n*-octanol. Determination was carried out at pH 7.4 in a mixture of equal volumes of buffer and *n*-octanol after shaking continuously for 18 h at room temperature. The concentration of complex in *n*-octanol was measured spectrophotometrically in order to determine values of D = [compound] (in *n*-octanol)/[compounds] (in buffer).^66,67^

## Supporting Information Available

Synthetic procedures, analytical data (PDF)
