# Supplementary material for: Metal(triphenylphosphine)-atovaquone Complexes: Synthesis, Antimalarial Activity, and Suppression of Heme Detoxification
Source: Inorg Chem. 2024 Aug 26;63(37):17087–99. doi: 10.1021/acs.inorgchem.4c02751 (PMC11409218; doi:10.1021/acs.inorgchem.4c02751)
Supplement: Supplementary file 1 — ic4c02751_si_001.pdf [file ic4c02751_si_001.pdf]

Supporting Information

Metal(triphenylphosphine)-atovaquone complexes: synthesis, antimalarial activity and suppression of heme detoxification

Luana Daniel[a,#], Arquímedes Karam[a,#], Chris Hebert J. Franco[b], Camila Conde[a], Adrielle Sacramento de Morais,[c] Joel Mosnier,[d,e,f,g] Isabelle Fonta,[d,e,f,g] Wilmer Villarreal,[h] Bruno Pradines[d,e,f,g], Diogo Rodrigo M. Moreira[c], Maribel Navarro[a,]*

[a] Laboratório de Química Bioinorganica e Catalise, Departamento Química, Instituto de Ciências Exatas, Universidade Federal de Juiz de Fora, Juiz de Fora, MG 36036-900, Brazil.

[b] Centro de Química Estrutural, Institute of Molecular Sciences, Instituto Superior Técnico, Universidade de Lisboa, Lisboa, 1049-001, Portugal.

[c] Instituto Gonçalo Moniz, FIOCRUZ, Salvador, BA 40296-710, Brazil.

[d] Unité Parasitologie et entomologie, Institut de recherche biomédicale des armées, Marseille, 13005, France.

[e] Aix-Marseille Univ, IRD, SSA, AP-HM, RITMES, Marseille, 13005, France.

[f] IHU Méditerranée Infection. Marseille, 13005, France.

[g] Centre National de Référence du Paludisme, Marseille, 13005, France.

[h] Grupo de Química Inorgânica Medicinal e Reações Aplicadas, Instituto de Química, Universidade Federal do Rio Grande do Sul, Porto Alegre, RS 91501-970, Brazil.

[#] Equally contributed to this work. *Corresponding author: M. Navarro (maribel.navarro@ufjf.br)

TABLE OF CONTENT

Figure S1- ^{1}H NMR spectrum of the Atovaquone in DMSO-d_6 4

Figure S2- $^{13}C\{^{1}H\}$ NMR spectrum of atovaquone in DMSO-d_6 4

Figure S3- Uv-Vis spectrum of atovaquone in DMSO 5

Figure S4- IR spectrum of atovaquone 5

Table S1- Molar conductivity measurements for the metal complexes **1**-**3.** 6

Figure S5- IR spectrum of [Ag(ATV)(PPh_3)] 6

Figure S6- IR spectrum of [Au(ATV)(PPh_3)]2H_2O. 7

Figure S7- IR spectrum of [Cu(ATV)(PPh_3)$_2$]. 7

Figure S8-^{1}H NMR spectrum of [Ag(ATV)(PPh_3)$_2$] (**1**) in DMSO-d_6 8

Figure S9- $^{13}C\{^{1}H\}$NMR spectrum of [Ag(ATV)(PPh_3)$_2$] (**1**) in DMSO-d_6 8

Figure S10- $^{31}P\{^{1}H\}$ NMR spectrum of [Ag(ATV)(PPh_3)$_2$] (**1**) in DMSO-d_6 9

Figure S11 - ^{1}H NMR spectrum of [Au(ATV)(PPh_3)].2H_2O (**2**) in DMSO-d_6 9

Figure S12- $^{13}C\{^{1}H\}$NMR spectrum of [Au(ATV)(PPh_3)].2H_2O (**2**) in DMSO-d_6 10

Figure S13 - $^{31}P\{^{1}H\}$NMR spectrum of [Au(ATV)(PPh_3)].2H_2O (**2**) in DMSO-d_6 10

Figure S14 - ^{1}H NMR spectra of [Cu(ATV)(PPh_3)$_2$] (**3**) in (A) dichlorometane-d_2; (B) DMSO-d_6 and (C) acetone-d_6 11

Figure S15 - $^{13}C\{^{1}H\}$NMR spectrum of [Cu(ATV)(PPh_3)$_2$] (**3**) in acetone-d_6 12

Figure S16 - $^{31}P\{^{1}H\}$NMR spectrum of [Cu(ATV)(PPh_3)$_2$] (**3**) in DMSO-d_6 12

Table S2- Crystal data and structure refinement of compounds **1**, **2**, and **3**. 13

Table S3- Selected geometric parameters (Å, °) for compound **1, 2** and **3** 14

Figure S17 - Crystal structure for compound [Ag(ATV)(PPh_3)$_2$]$_2$ (**1**). Thermal ellipsoids were drawn with 25% of probability and H-atoms were omitted for clarity. The units have similar parameters and the geometry in addition to *β* angle shows a value close to 90°, which raised the question whether this is really a crystalline structure with a non-centrosymmetric space group or there might be an inversion center between the units, leading to the formation of a group with higher symmetry[1]. To this end, Laue symmetry was inspected for evidence. Diffractometer software suggests a non-centrosymmetric space group and the PLATON ADDSYM routine[2] does not display any additional symmetry elements (no obvious space group change) 15

Figure S18 - Crystal structure of compound [Au(ATV)(PPh_3)].H_2O **(2)**. Water molecules present a positional disorder with a 64:36 contribution. Thermal ellipsoids were drawn with 25% of probability and H-atoms were omitted for clarity. 15

Figure S19 - Crystal structure of compound [Cu(ATV)(PPh_3)$_2$] **(3).** Thermal ellipsoids were drawn with 25% of probability and H-atoms were omitted for clarity 16

Figure S20- Stability study by **^{1}H NMR** of [Ag(ATV)(PPh_3)] (**1**) in DMSO-d_6 16

Figure S21 - Stability study by **$^{31}P\{^{1}H\}$ NMR** of [Ag(ATV)(PPh_3)] (**1**) in DMSO-d_6 17

Figure S22 - Stability study by **^{1}H NMR** of [Au(ATV)(PPh_3)].2H_2O (**2**) in DMSO-d_6 17

Figure S24 - Stability study by **^{1}H NMR** of [Cu(ATV)(PPh_3)$_2$] (**3**) in DMSO-d_6 18

Figure S26 - Stability study by Uv-Vis of [Ag(ATV)(PPh_3)] (**1**) in DMSO 19

Figure S27 - Stability study by UV-vis of [Au(ATV)(PPh_3)].2H_2O (**2**) in DMSO 20

Figure S28 - Stability study by UV-Vis of [Cu(ATV)(PPh_3)$_2$] (**3**) in DMSO 20

Figure S29- Stability study by Uv-Vis of [Ag(ATV)(PPh_3)$_2$] (**1**) in solution of 90% DMSO and 10% water 21

Figure S31- Stability study by Uv-Vis of [Cu(ATV)(PPh_3)$_2$] (**3**) in solution of 90% DMSO and 10% water 22

Figure S33 - Stability study by Uv-Vis of [Ag(ATV)(PPh_3)$_2$] (**1**) in solution of 90% DMSO and 10% RPMI culture medium 23

Figure S35- Stability study by Uv-Vis of [Cu(ATV)(PPh_3)$_2$] (**3**) in solution of 90% DMSO and 10% RPMI culture medium 24

Figure S37- Titration of ferriprotoporphyrin IX [Fe(III)-PPIX, hemin]. Arrow indicates the decrease in absorbance upon increasing concentration of compounds [Au(ATV)(PPh_3)].2H_2O (**2**) 25

Figure S38- Titration of ferriprotoporphyrin IX [Fe(III)-PPIX, hemin]. Arrow indicates the decrease in absorbance upon increasing concentration of compounds [Cu(ATV)(PPh_3)$_2$] (**3**) 25

Figure S39- Spectra ß-hematin formation. 26

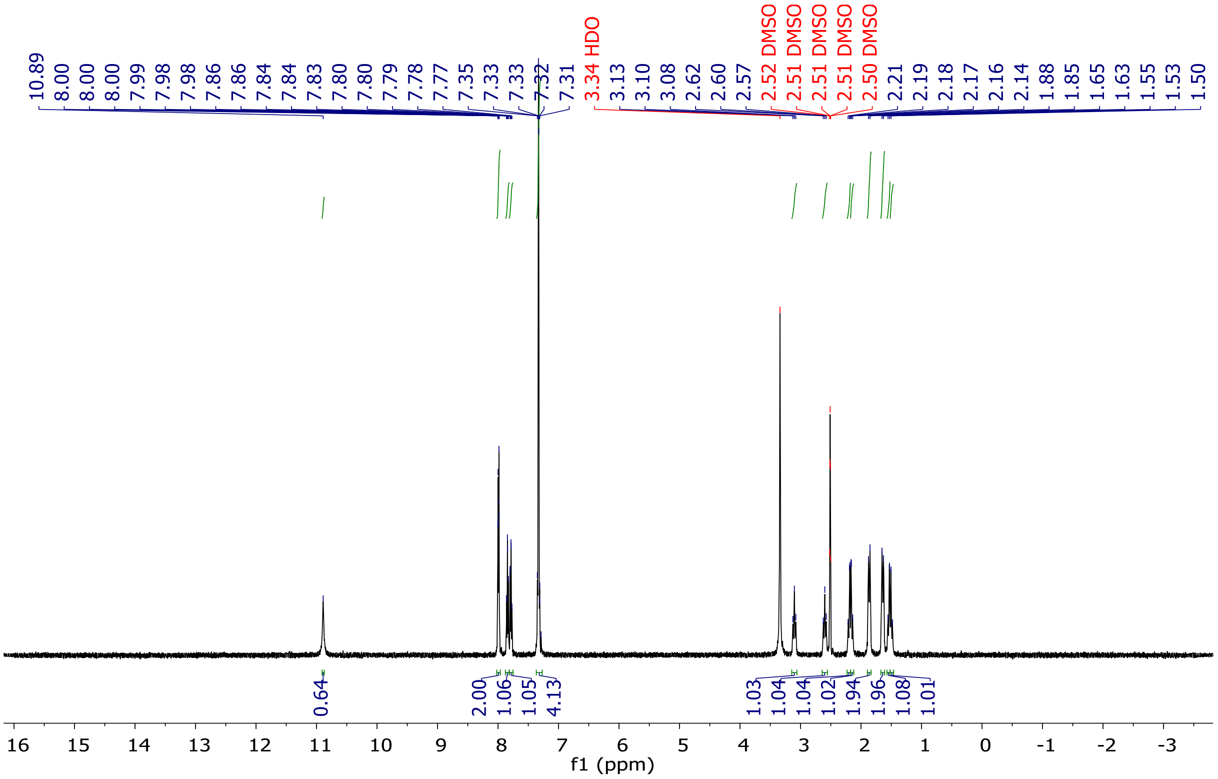

Figure S1- ^{1}H NMR spectrum of the Atovaquone in DMSO-d_6

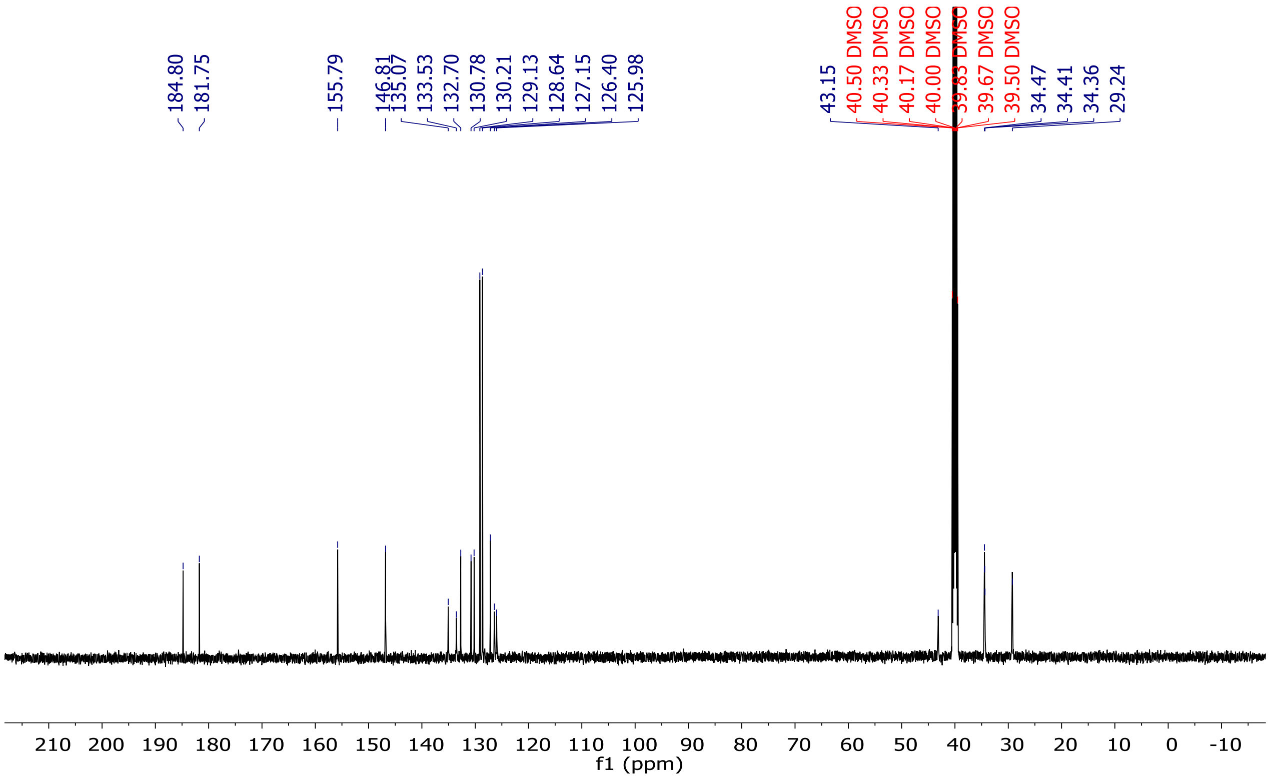

Figure S2- ^{13}C{^{1}H} NMR spectrum of atovaquone in DMSO-d_6

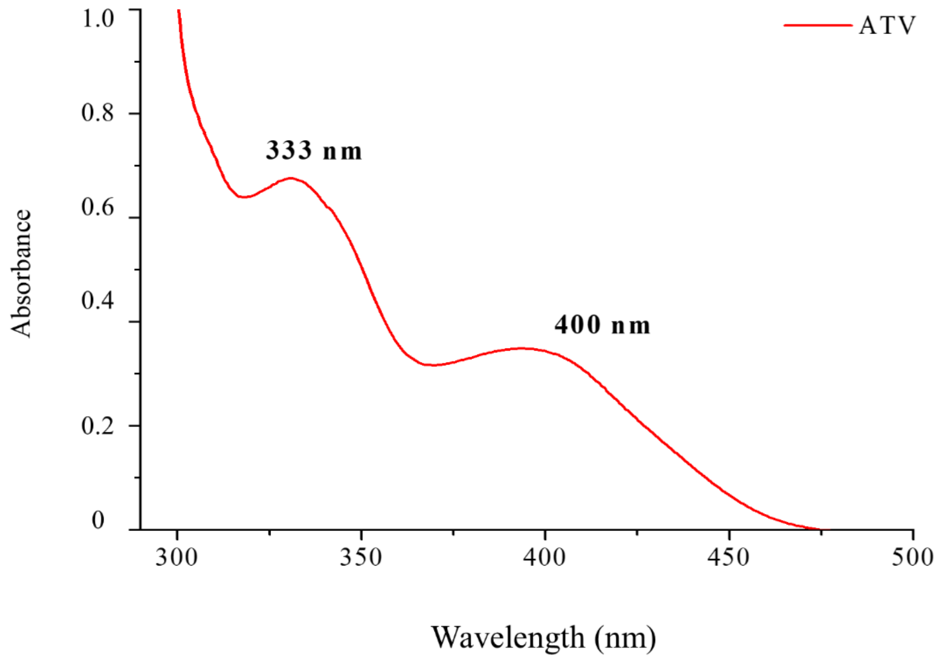

Figure S3- Uv-Vis spectrum of atovaquone in DMSO

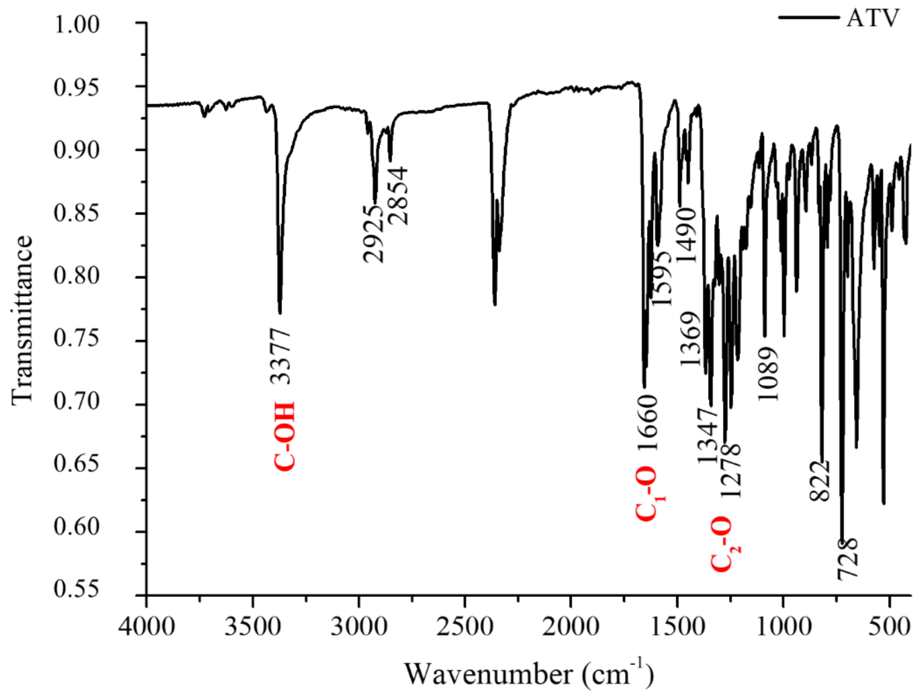

Figure S4- IR spectrum of atovaquone.

Table S1- Molar conductivity measurements for the metal complexes **1-3.**

**Compound**	Λ_M **(DMSO)** **(Ohm^{-1}cm^{2}mol^{-1})** **Day 1**	Λ_M **(DMSO)** **(Ohm^{-1}cm^{2}mol^{-1})** **Day 2**	Λ_M **(DMSO)** **(Ohm^{-1}cm^{2}mol^{-1})** **Day 3**	Λ_M **(DMSO)** **(Ohm^{-1}cm^{2}mol^{-1})** **Day 7**	Λ_M **(DMSO)** **(Ohm^{-1}cm^{2}mol^{-1})** **Day 15**
[Au(ATV)(PPh_3)].2H_2O	12.69	14.51	12.53	12.41	11.84
[Ag(ATV)(PPh_3)$_2$]	12.81	13.90	16.14	13.70	12.17
[Cu(ATV)(PPh_3)$_2$]	3.71	3.80	3.47	3.14	3.05
Atovaquone	0.93	1.07	1.23	1.68	2.04

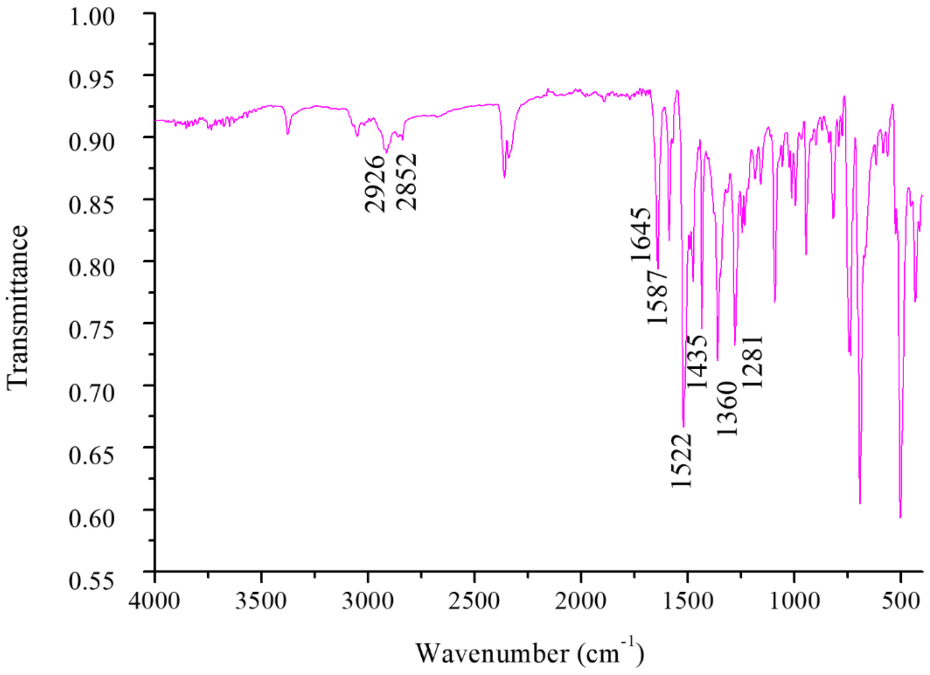

Figure S5- IR spectrum of [Ag(ATV)(PPh_3)].

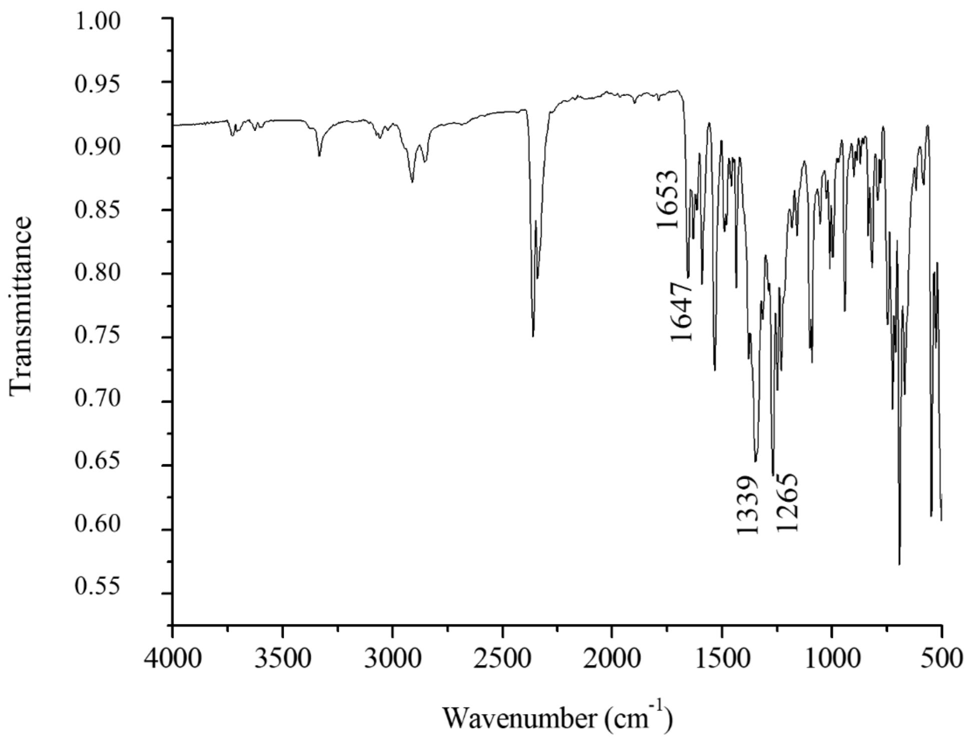

Figure S6- IR spectrum of [Au(ATV)(PPh_3)]$2H_2O$.

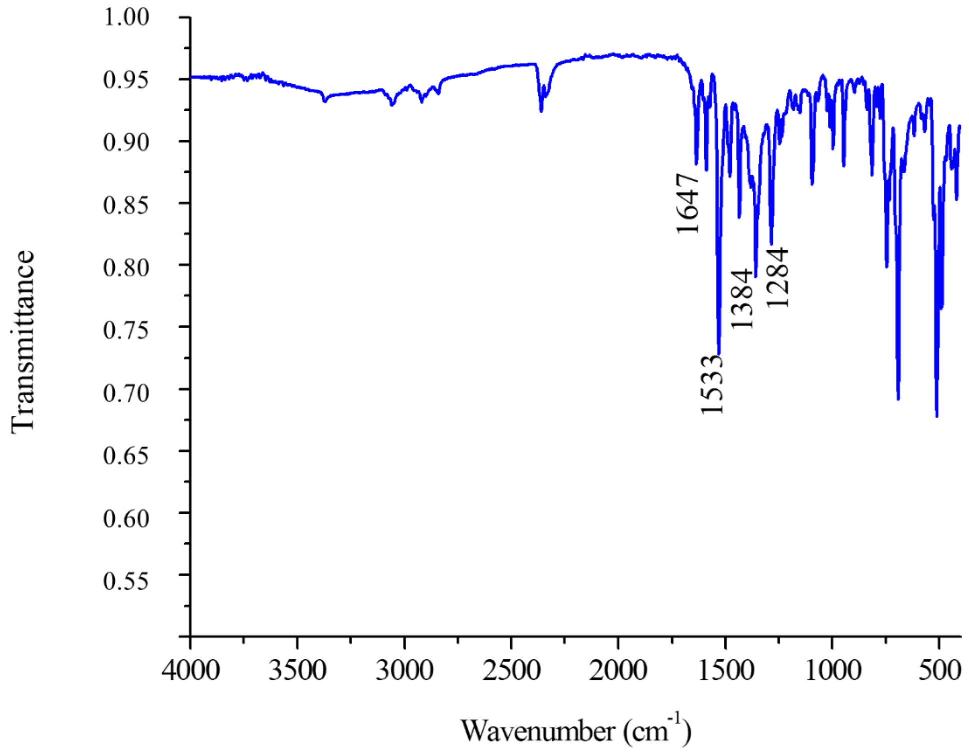

Figure S7- IR spectrum of [Cu(ATV)(PPh_3)$_2$].

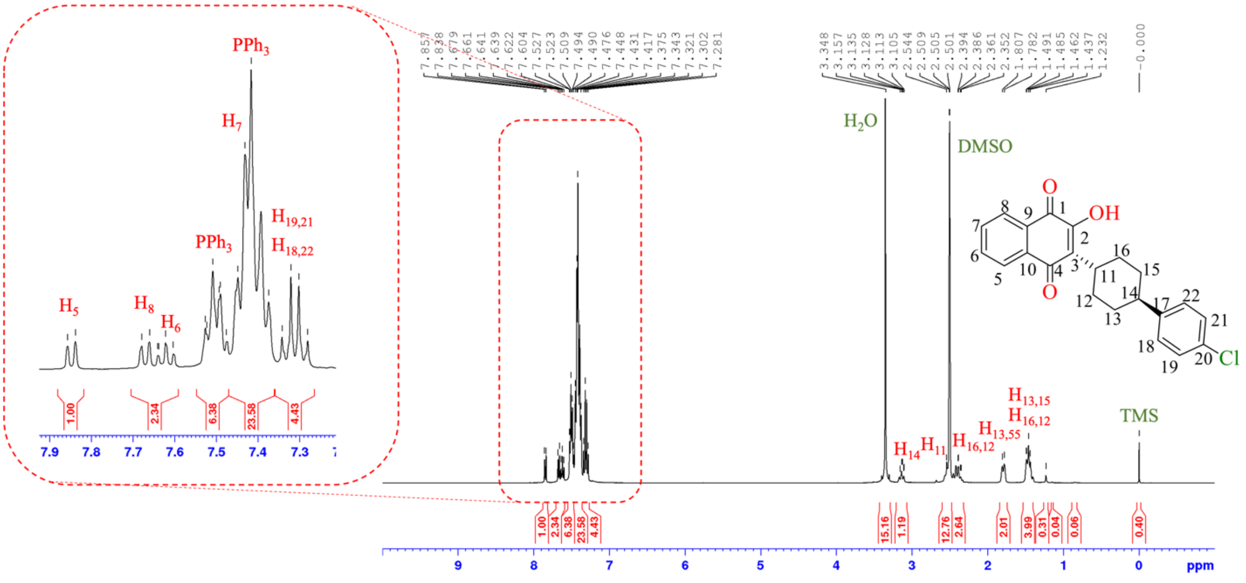

Figure S8-^{1}H NMR spectrum of $[Ag(ATV)(PPh_3)_2]$ (**1**) in DMSO-*d_6*

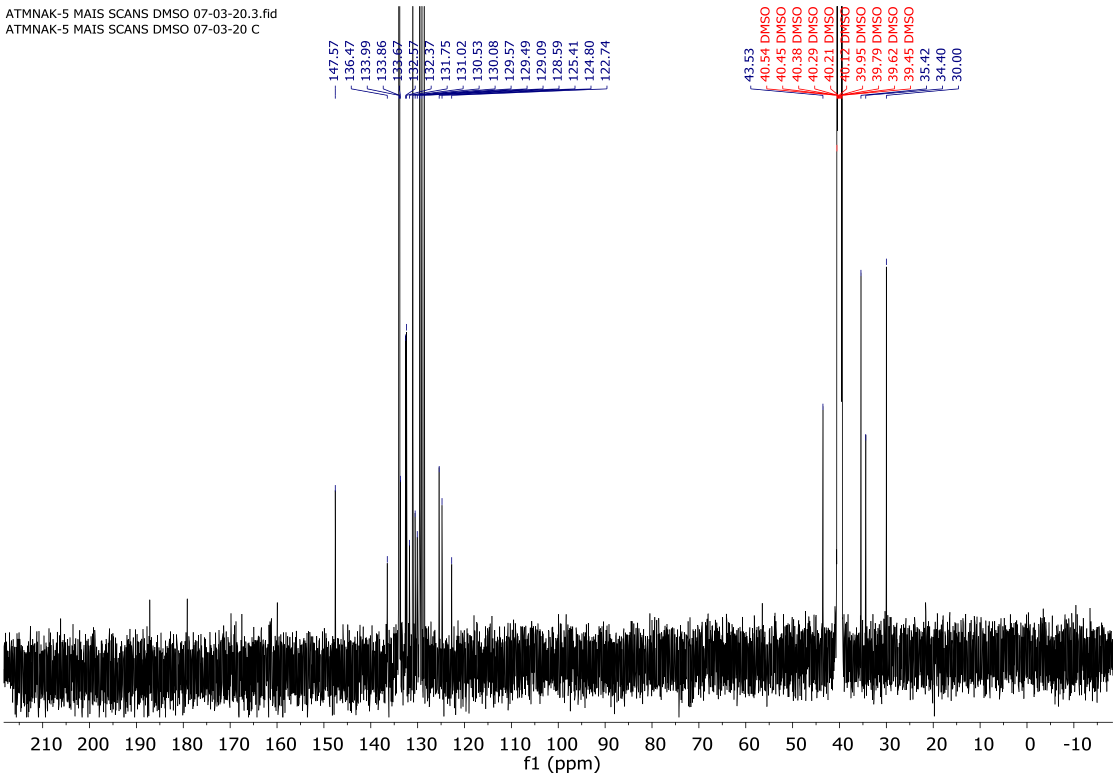

Figure S9- $^{13}C\{^{1}H\}$NMR spectrum of $[Ag(ATV)(PPh_3)_2]$ (**1**) in DMSO-*d_6*

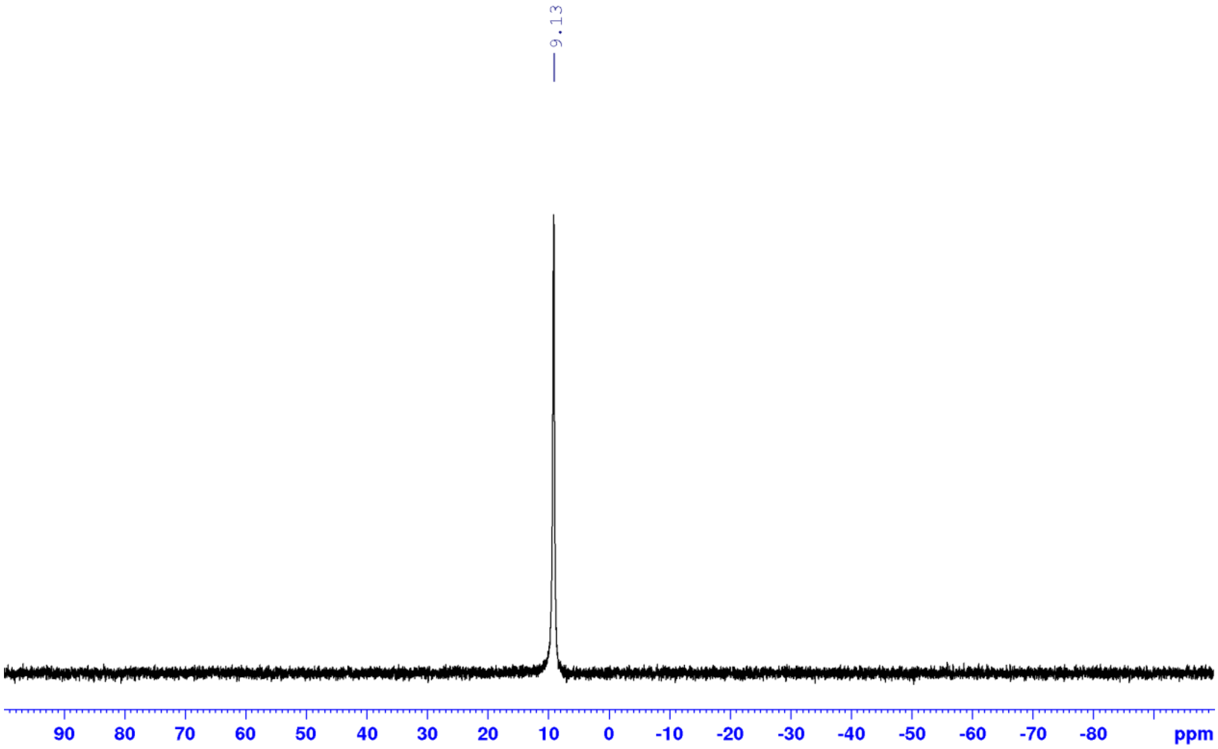

Figure S10- $^{31}P\{^{1}H\}$ NMR spectrum of $[Ag(ATV)(PPh_3)_2]$ (**1**) in DMSO-d_6

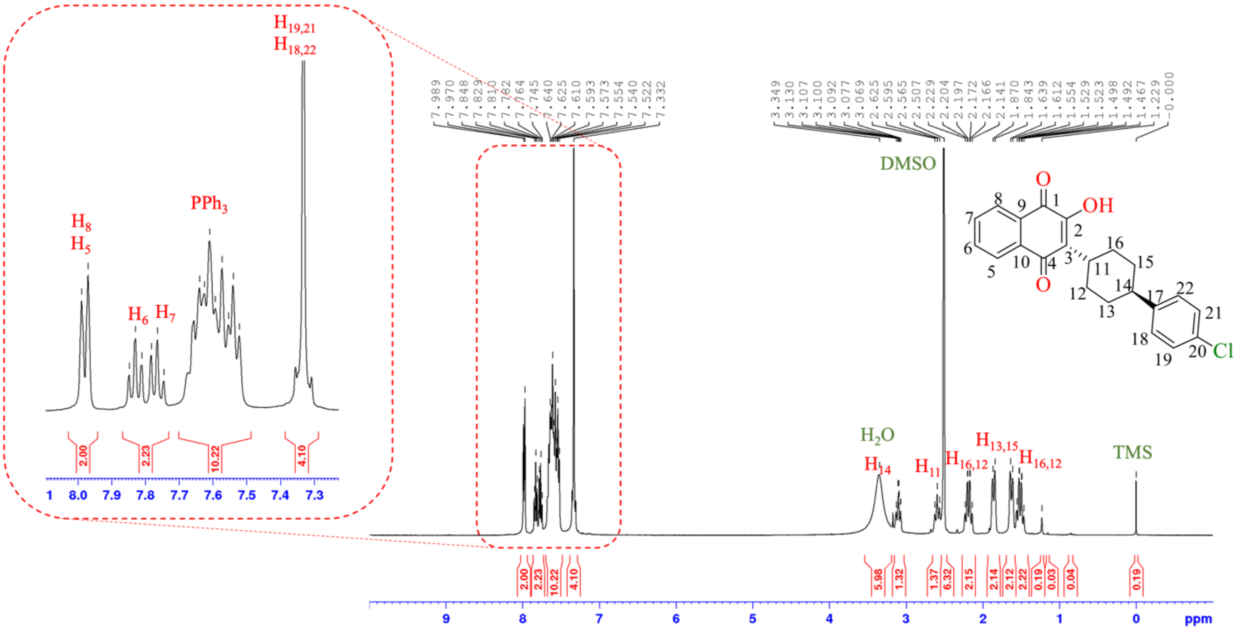

Figure S11 - ^{1}H NMR spectrum of $[Au(ATV)(PPh_3)].2H_2O$ (**2**) in DMSO-d_6

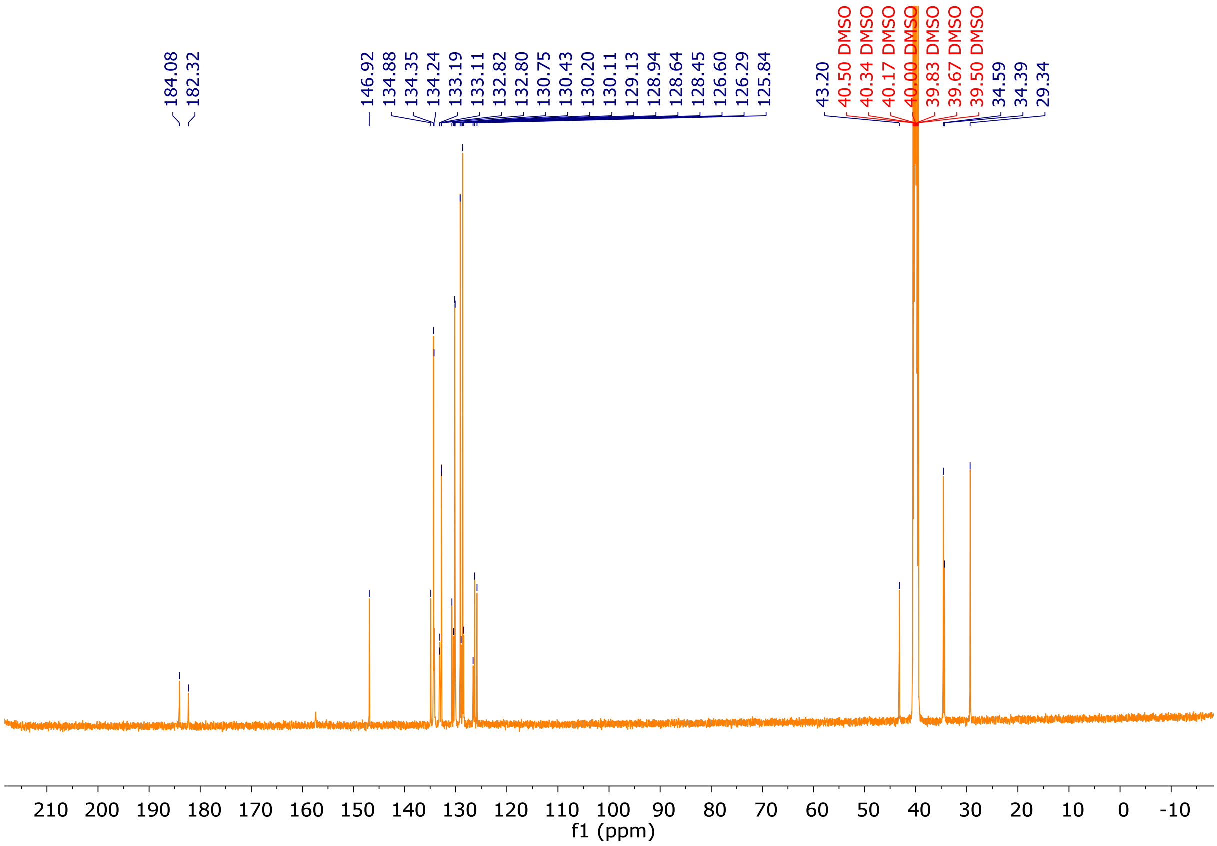

Figure S12- $^{13}C\{^{1}H\}$NMR spectrum of [Au(ATV)(PPh$_3$)].2H$_2$O (**2**) in DMSO-*d*$_6$

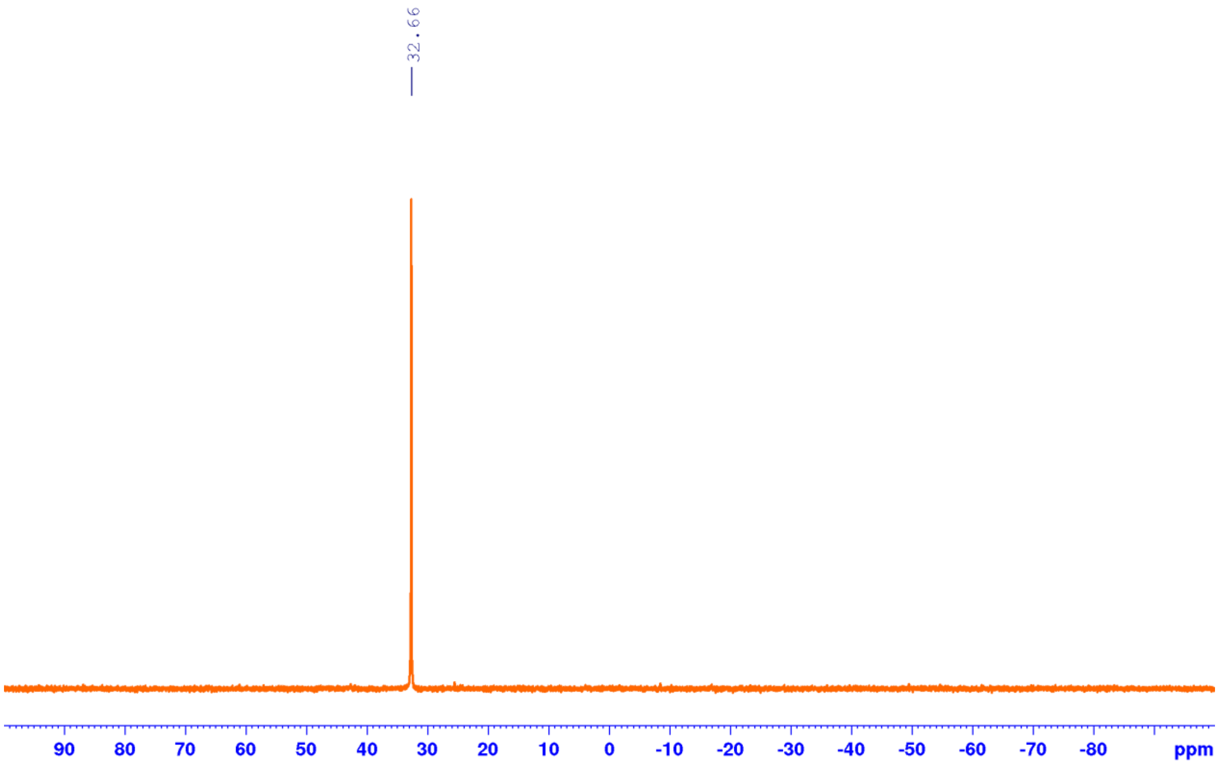

Figure S13 - $^{31}P\{^{1}H\}$NMR spectrum of [Au(ATV)(PPh$_3$)].2H$_2$O (**2**) in DMSO-*d*$_6$

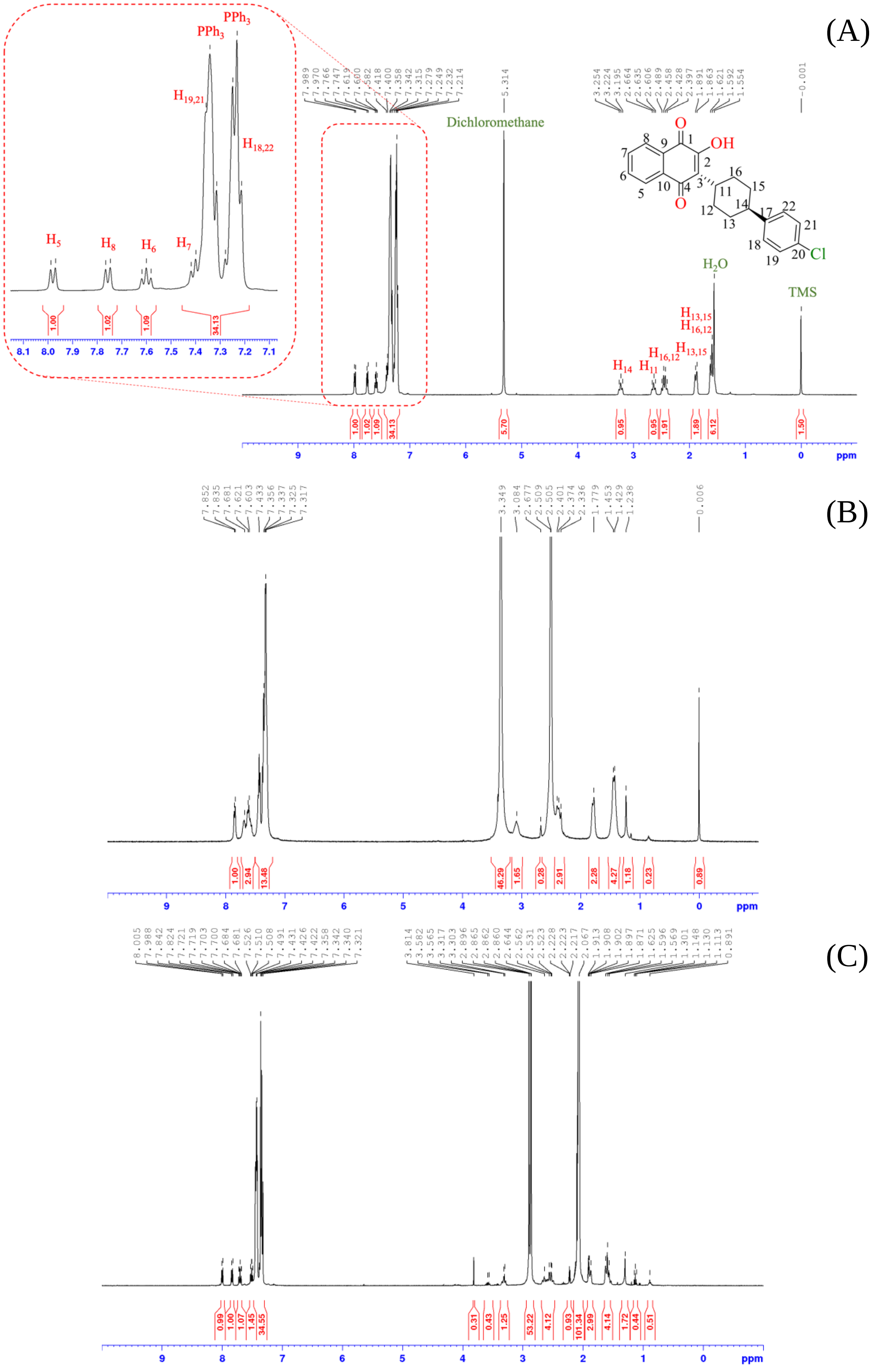

Figure S14 - ^{1}H NMR spectra of [Cu(ATV)(PPh3)2] (**3**) in (A) dichlorometane-d_2; (B) DMSO-d_6 and (C) acetone-d_6

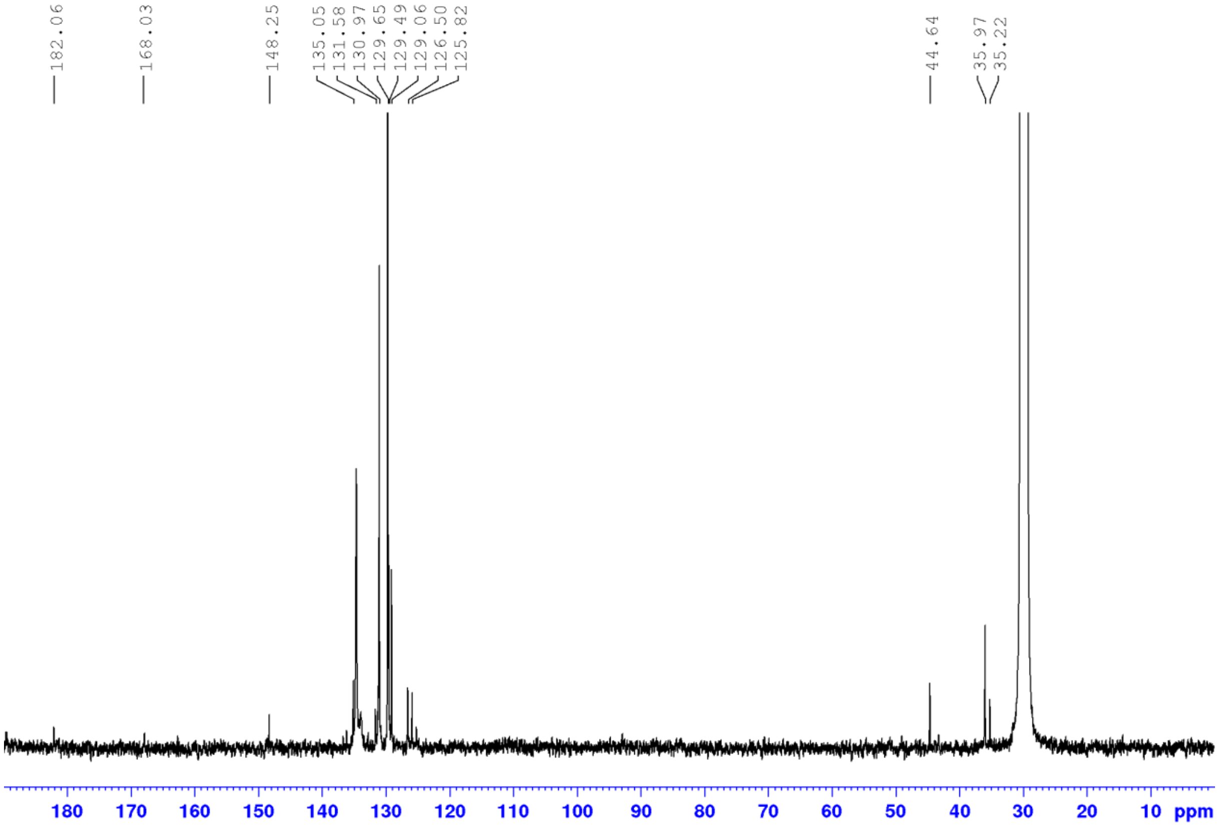

Figure S15 - $^{13}C\{^1H\}$NMR spectrum of [Cu(ATV)(PPh_3)$_2$] (**3**) in acetone-d_6

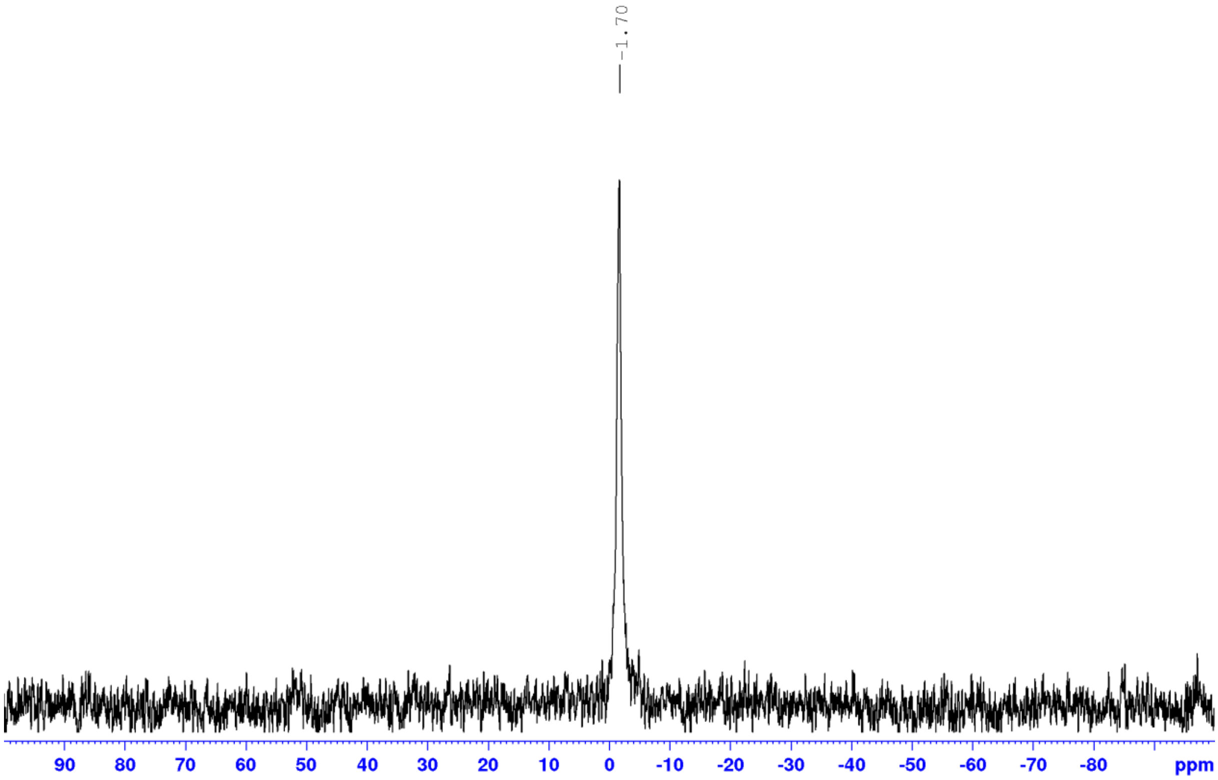

Figure S16 - $^{31}P\{^1H\}$NMR spectrum of [Cu(ATV)(PPh_3)$_2$] (**3**) in DMSO-d_6

Table S2- Crystal data and structure refinement of compounds **1**, **2**, and **3**.

Crystal data	1	2	3
Mr	1996.44	841.05	953.89
Crystal system, space group	Monoclinic, P*2₁*	Orthorhombic, P*222₁*	Triclinic, P-*1*
Temperature (K)	291	292	291
a, b, c (Å)	9.13036 (18), 39.6718 (10), 13.5519 (3)	11.6627 (3), 12.2132 (4), 25.2344 (7)	9.1894 (4), 14.4657 (8), 19.6569 (10)
α, β, γ (°)	90, 92.694 (2), 90	90, 90, 90	71.871 (5), 81.878 (4), 86.042 (4)
V ($Å^3$)	4903.33 (19)	3594.36 (18)	2457.5 (2)
Z	2	4	2
Radiation type	CuKα	MoKα	CuKα
μ (mm^{-1})	4.76	4.25	2.09
Crystal size (mm)	0.49 × 0.08 × 0.06	0.25 × 0.19 × 0.07	0.73 × 0.05 × 0.03
No. of measured, independent and observed [I > 2σ(I)] reflections	25509, 6484, 6237	41962, 6599, 5903	15067, 2839, 2380
Rint	0.087	0.054	0.172
(sin θ/λ)max ($Å^{-1}$)	0.431	0.602	0.410
R[F2 > 2σ(F^2)], wR(F^2), S	0.061, 0.157, 1.00	0.036, 0.070, 1.08	0.077, 0.209, 1.03
No. of reflections	6484	6599	2839
No. of parameters	1171	424	587
No. of restraints	1	0	0
Δρmax, Δρmin ($eÅ^{-3}$)	0.58, −0.65	1.36, −0.60	0.61, −0.59

Table S3- Selected geometric parameters (Å, °) for compound **1, 2** and **3**

**Compound 1**			
Distances			
Ag1—P1	2.444 (6)	Ag2—P3	2.428 (6)
Ag1—P2	2.434 (6)	Ag2—P4	2.423 (6)
Ag1—O1	2.558 (17)	Ag2—O5	2.570 (17)
Ag1—O2	2.303 (16)	Ag2—O4	2.325 (16)
Angles			
P1—Ag1—O1	101.5 (4)	P3—Ag2—O5	107.0 (4)
P2—Ag1—P1	132.3 (2)	P4—Ag2—P3	129.3 (2)
P2—Ag1—O1	107.3 (4)	P4—Ag2—O5	104.9 (4)
O2—Ag1—P1	113.9 (4)	O4—Ag2—P3	108.4 (4)
O2—Ag1—P2	111.9 (4)	O4—Ag2—P4	120.3 (4)
O2—Ag1—O1	67.5 (6)	O4—Ag2—O5	65.9 (6)
**Compound 2**			
Distances			
Au1—Au1^{i}	3.2547 (7)	O1—C14	1.289 (9)
Au1—P1	2.200 (2)	O2—C15	1.201 (10)
Au1—O1	2.074 (5)		
Angles			
O1—Au1—P1	179.54 (19)		
Symmetry code: ***(i)*** *x, −y+1, −z+1.*			
**Compound 3**			
Distances			
Cu1—P4	2.229 (2)	Cu1—O2	2.027 (6)
Cu1—P3	2.232 (2)	Cu1—O1	2.215 (6)
Angles			
P4—Cu1—P3	124.24 (9)	O2—Cu1—O1	77.0 (3)
O2—Cu1—P4	113.94 (16)	O1—Cu1—P4	107.70 (15)
O2—Cu1—P3	119.40 (16)	O1—Cu1—P3	98.92 (16)

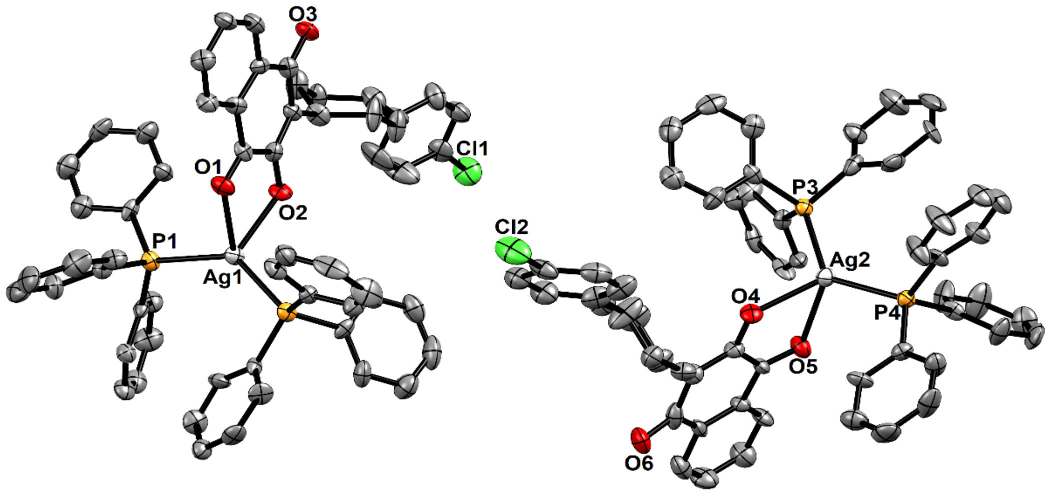

Figure S17 - Crystal structure for compound $[Ag(ATV)(PPh_3)_2]_2$ (**1**). Thermal ellipsoids were drawn with 25% of probability and H-atoms were omitted for clarity. The units have similar parameters and the geometry in addition to β angle shows a value close to 90°, which raised the question whether this is really a crystalline structure with a non-centrosymmetric space group or there might be an inversion center between the units, leading to the formation of a group with higher symmetry[1]. To this end, Laue symmetry was inspected for evidence. Diffractometer software suggests a non-centrosymmetric space group and the PLATON ADDSYM routine[2] does not display any additional symmetry elements (no obvious space group change)

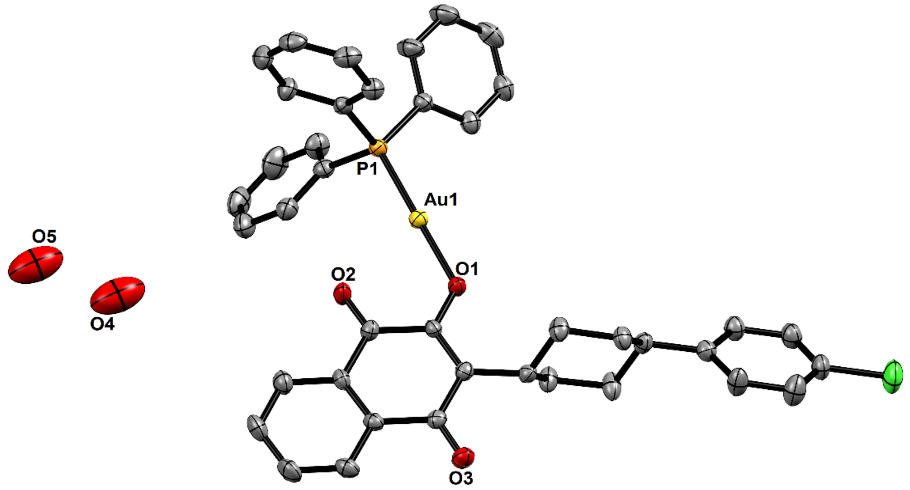

Figure S18 - Crystal structure of compound $[Au(ATV)(PPh_3)].H_2O$ **(2)**. Water molecules present a positional disorder with a 64:36 contribution. Thermal ellipsoids were drawn with 25% of probability and H-atoms were omitted for clarity.

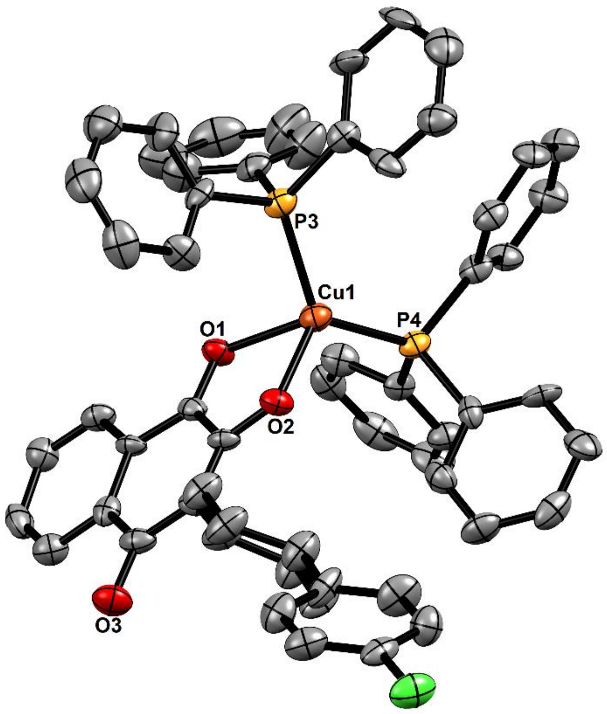

Figure S19 - Crystal structure of compound [Cu(ATV)(PPh_3)$_2$] **(3).** Thermal ellipsoids were drawn with 25% of probability and H-atoms were omitted for clarity

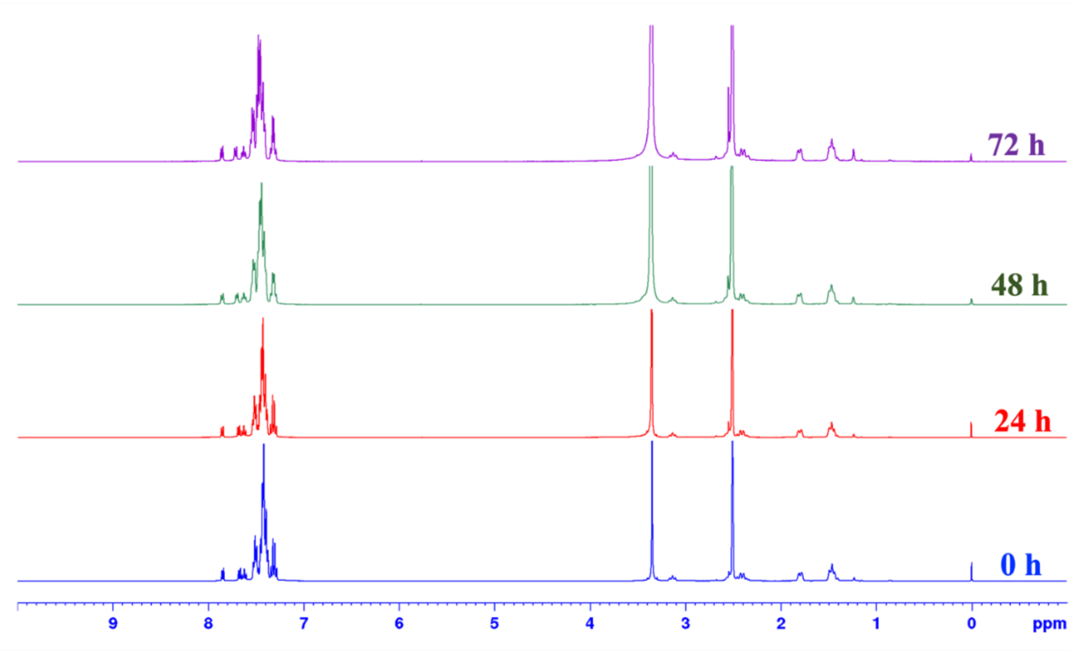

Figure S20- Stability study by **^{1}H NMR** of [Ag(ATV)(PPh_3)] (**1**) in DMSO-d_6

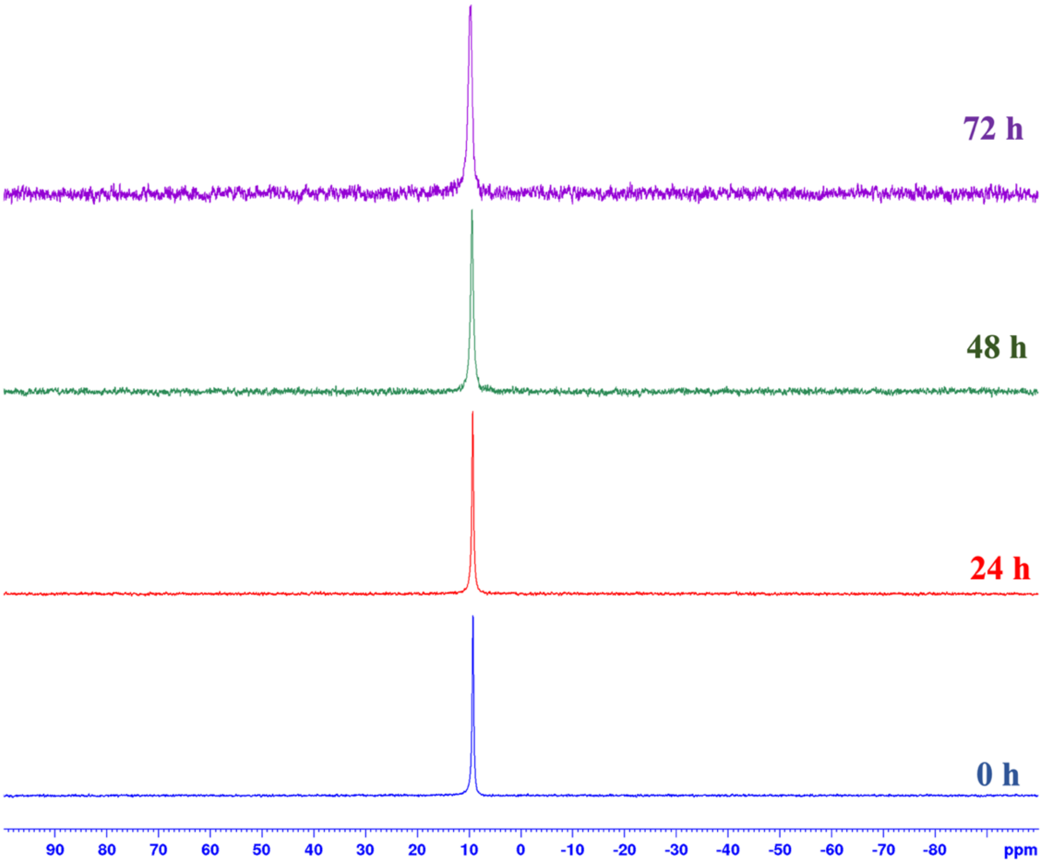

Figure S21 - Stability study by ^{31}**P{^{1}H} NMR** of [Ag(ATV)(PPh_3)] (**1**) in DMSO-d_6

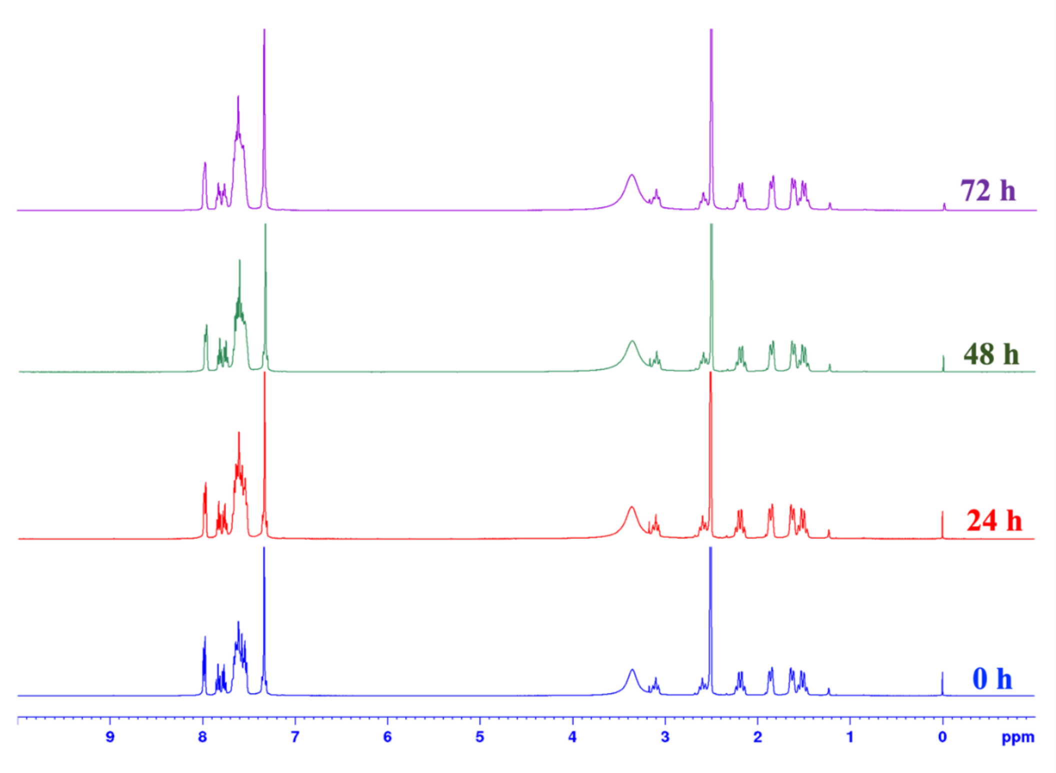

Figure S22 - Stability study by ^{1}**H NMR** of [Au(ATV)(PPh_3)].2H_2O (**2**) in DMSO-d_6

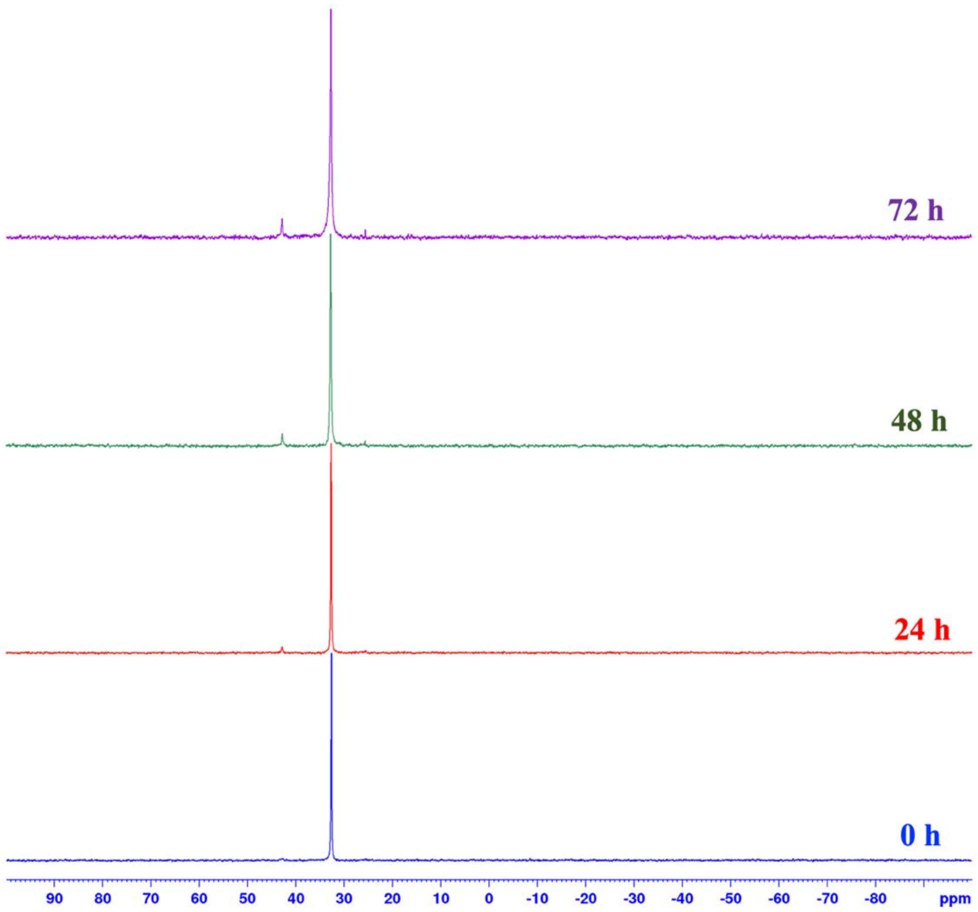

Figure S23- Stability study by **^{31}P{^{1}H} NMR** of [Au(ATV)(PPh_3)].2H_2O (**2**) in DMSO-d_6

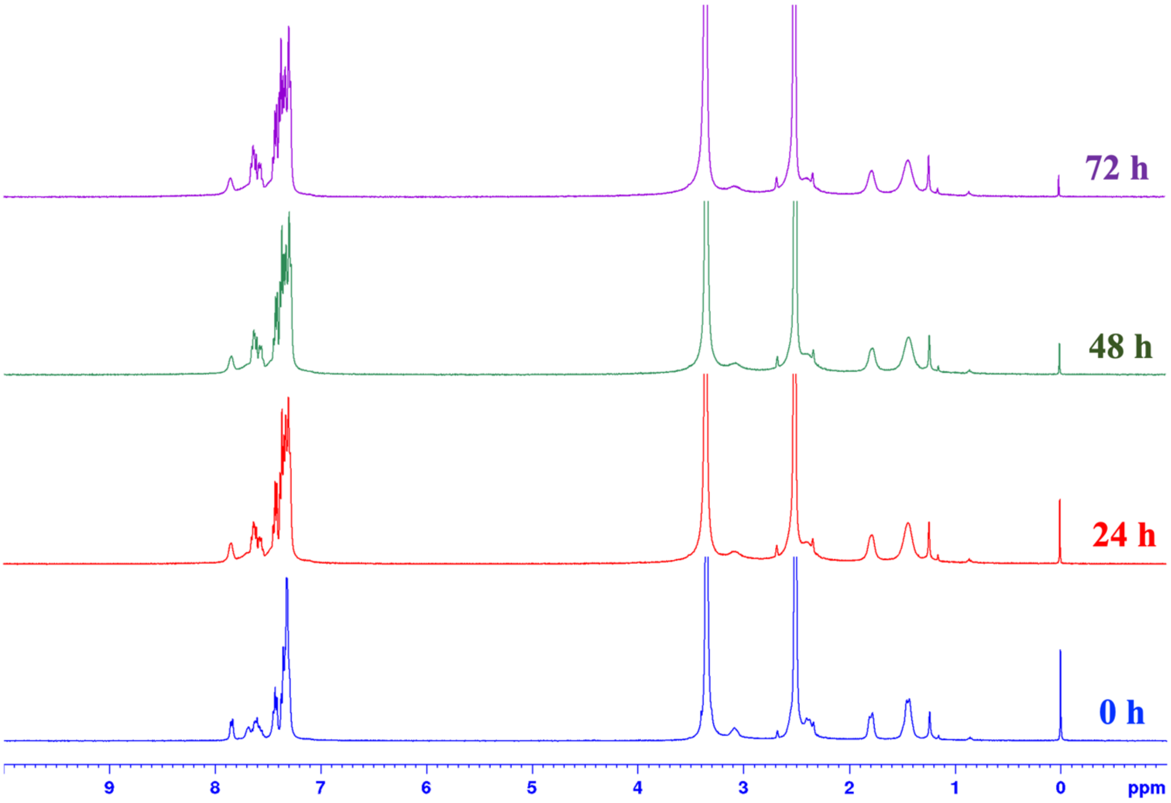

Figure S24 - Stability study by **^{1}H NMR** of [Cu(ATV)(PPh_3)$_2$] (**3**) in DMSO-d_6

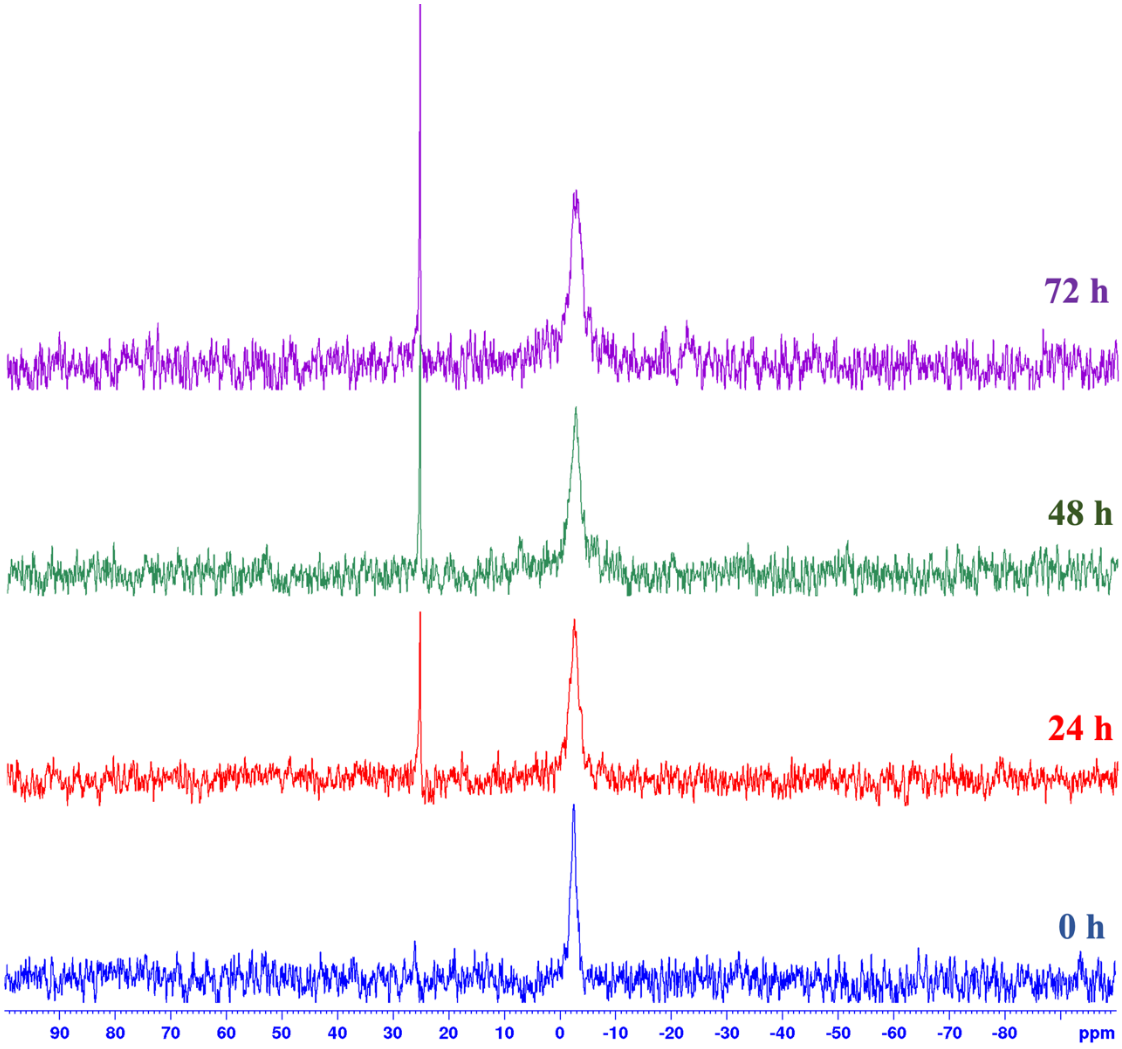

Figure S25 - Stability study by **^{31}P{^{1}H} NMR** of [Cu(ATV)(PPh_3)$_2$] (**3**) in DMSO-d_6

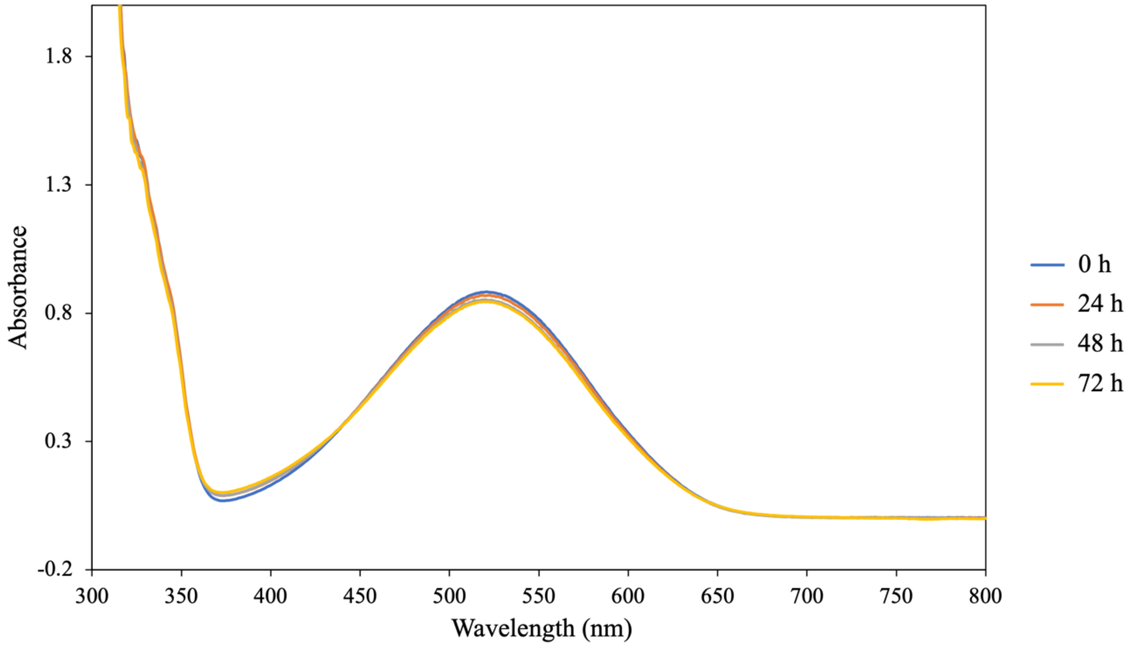

Figure S26 - Stability study by Uv-Vis of [Ag(ATV)(PPh_3)] (**1**) in DMSO

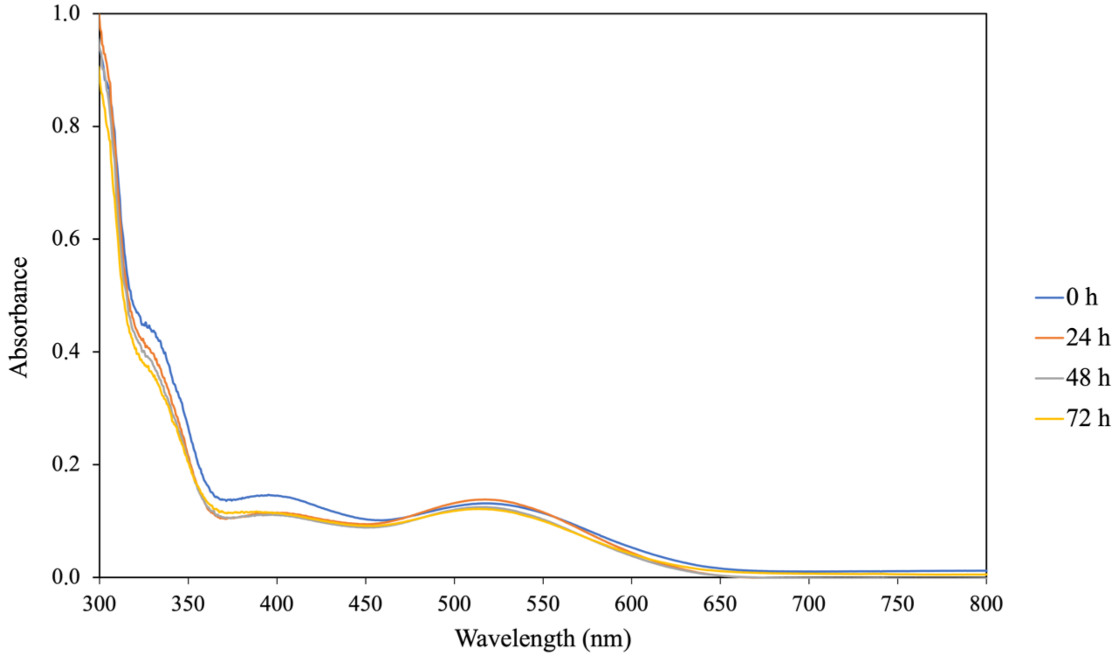

Figure S27 - Stability study by UV-vis of [Au(ATV)(PPh_3)].2H_2O (**2**) in DMSO

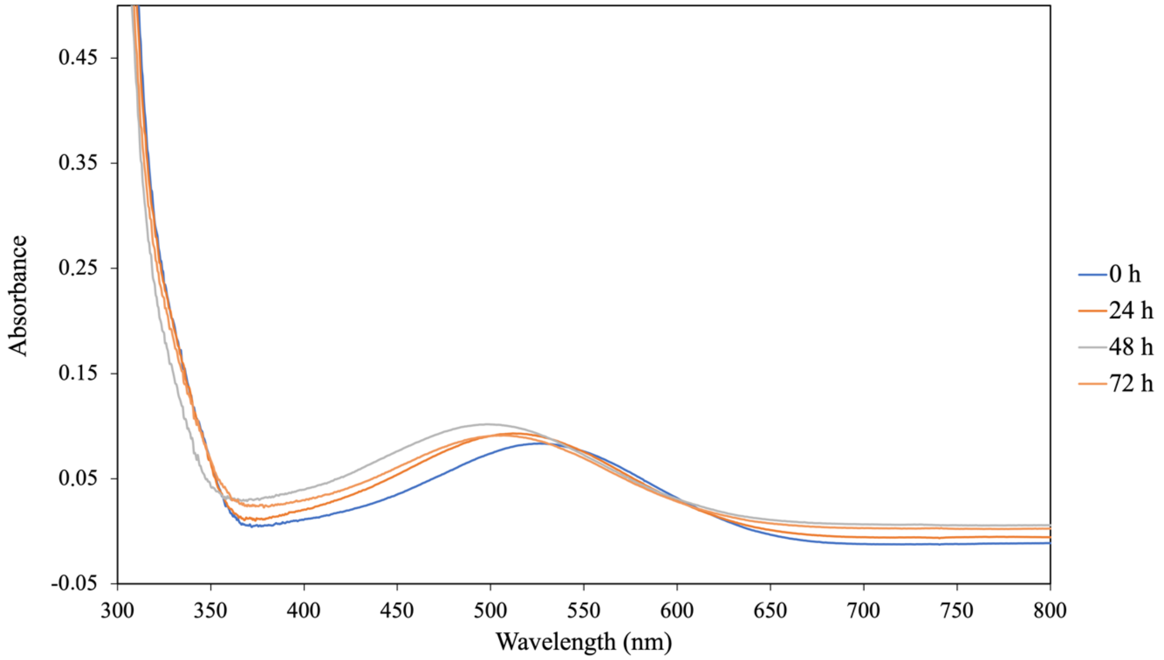

Figure S28 - Stability study by UV-Vis of [Cu(ATV)(PPh_3)$_2$] (**3**) in DMSO

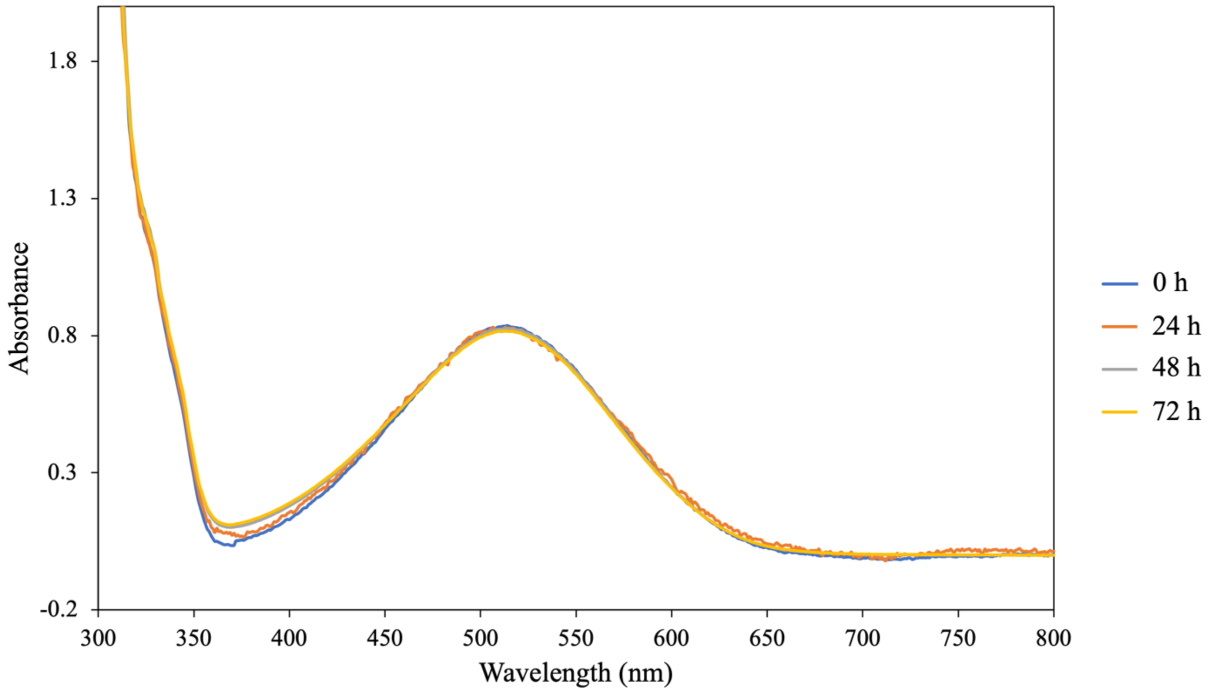

Figure S29- Stability study by Uv-Vis of [Ag(ATV)(PPh_3)$_2$] (**1**) in solution of 90% DMSO and 10% water

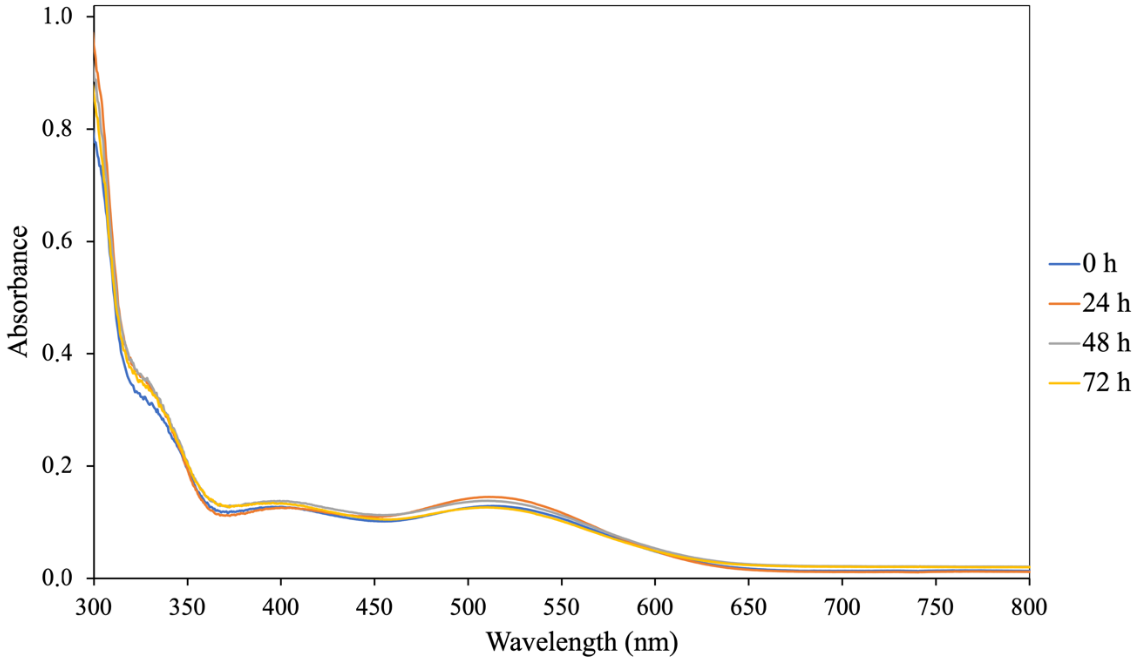

Figure S30- Stability study by Uv-Vis of [Au(ATV)(PPh_3)] (**2**) in solution of 90% DMSO and 10% water

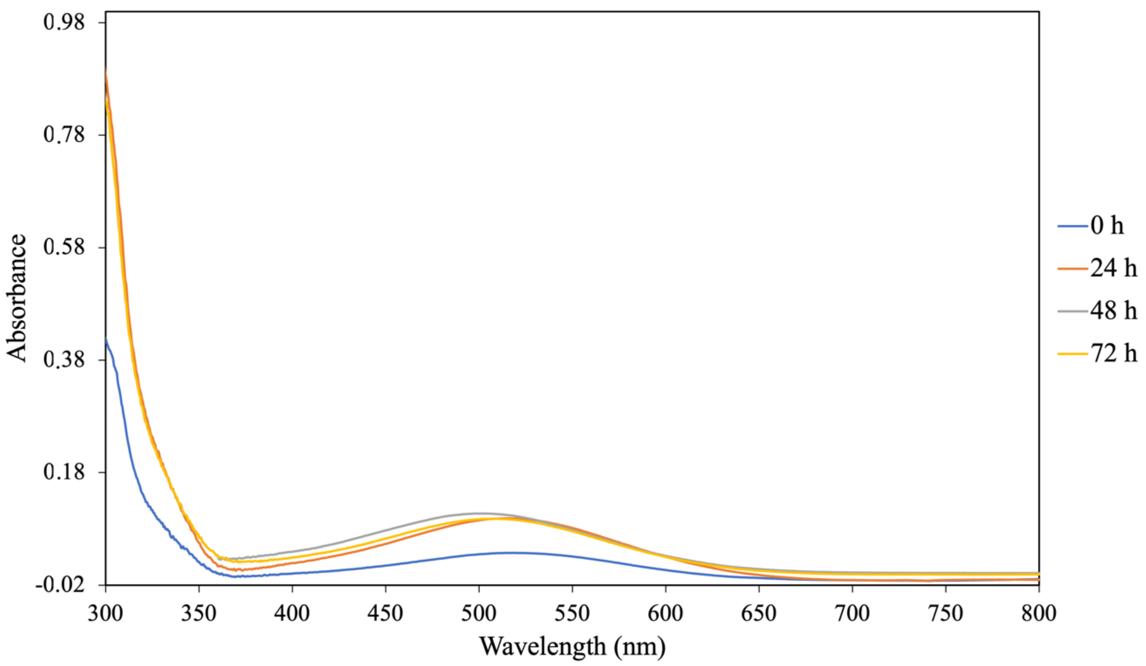

Figure S31- Stability study by Uv-Vis of [Cu(ATV)(PPh$_3$)$_2$] (**3**) in solution of 90% DMSO and 10% water

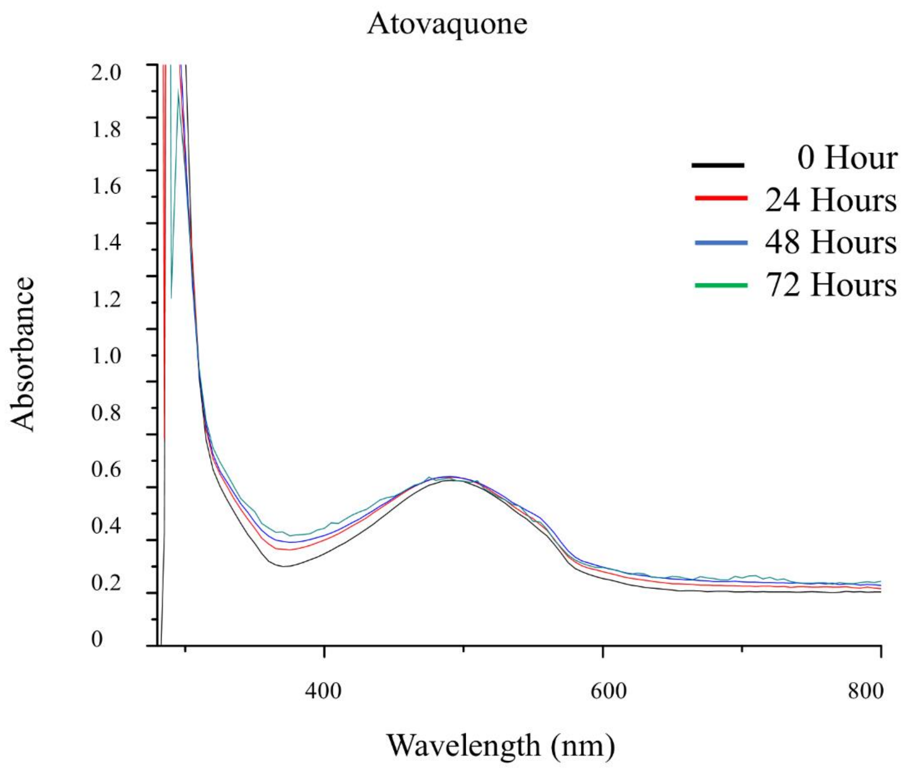

Figure S32- Stability study by Uv-Vis of Atovaquone in solution of 5% DMSO and 95% RPMI culture medium

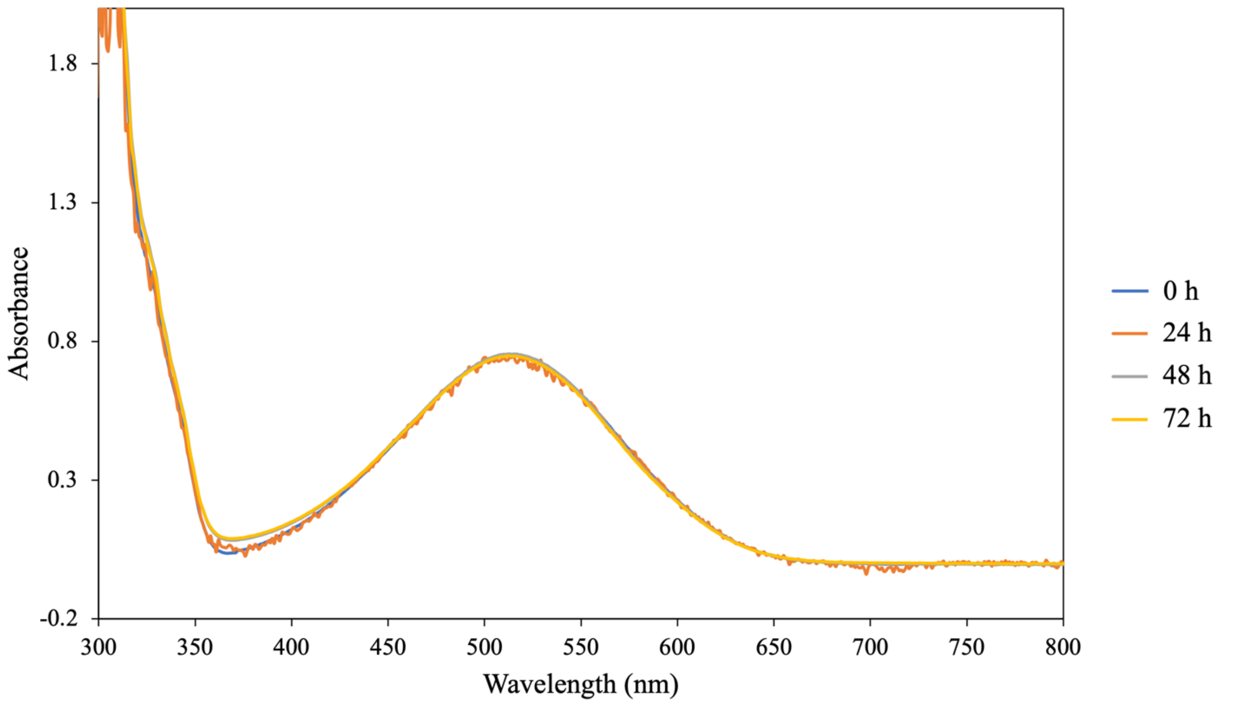

Figure S33 - Stability study by Uv-Vis of $[Ag(ATV)(PPh_3)_2]$ (**1**) in solution of 90% DMSO and 10% RPMI culture medium

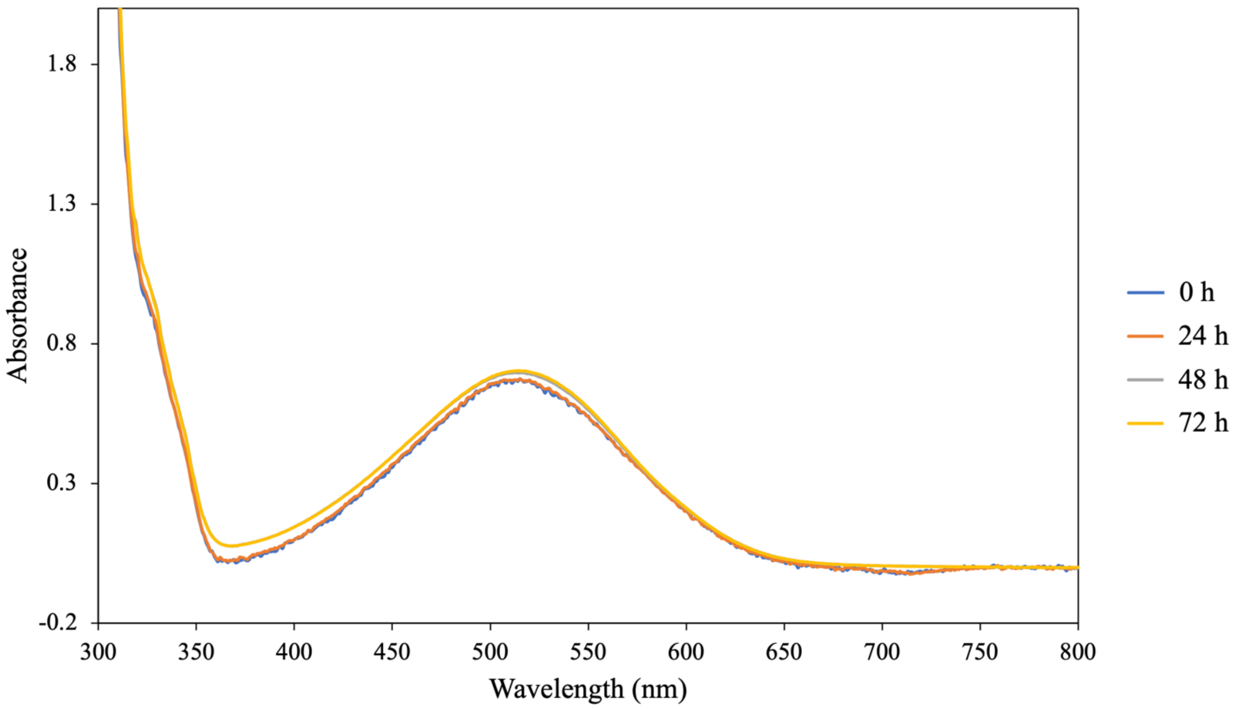

Figure S34- Stability study by Uv-Vis of $[Au(ATV)(PPh_3)].2H_2O$ (**2**) in solution of 90% DMSO and 10% RPMI culture medium

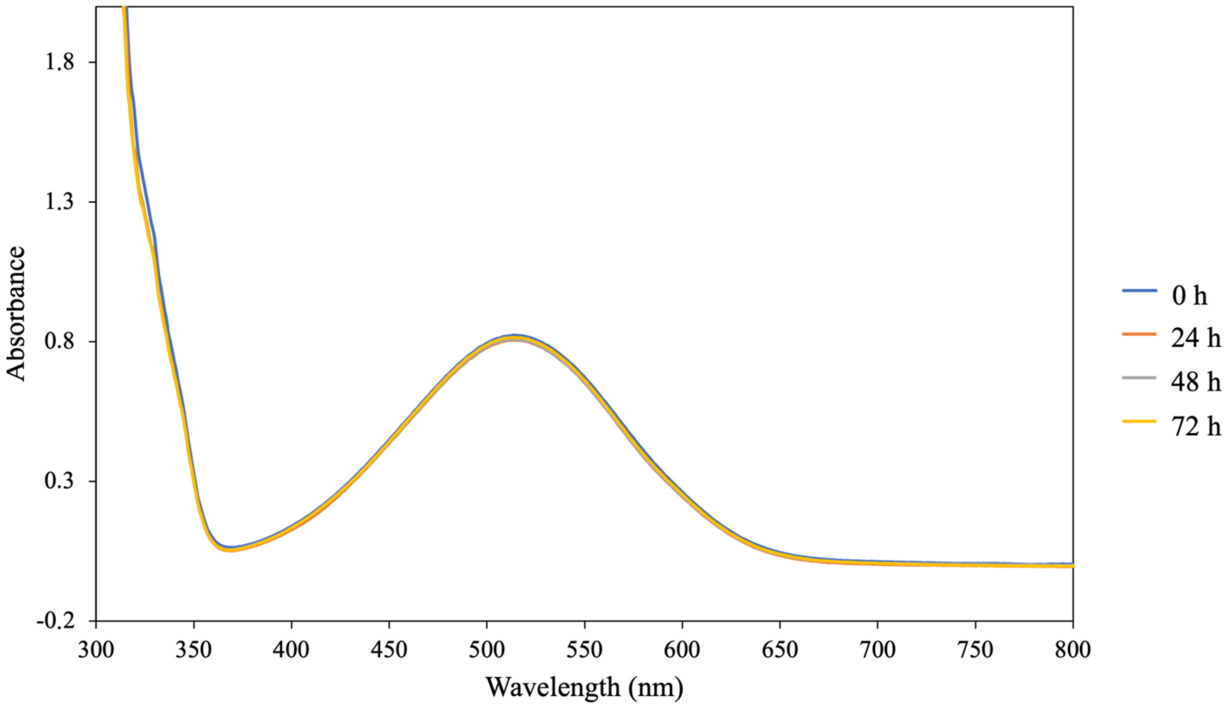

Figure S35- Stability study by Uv-Vis of [Cu(ATV)(PPh_3)$_2$] (**3**) in solution of 90% DMSO and 10% RPMI culture medium

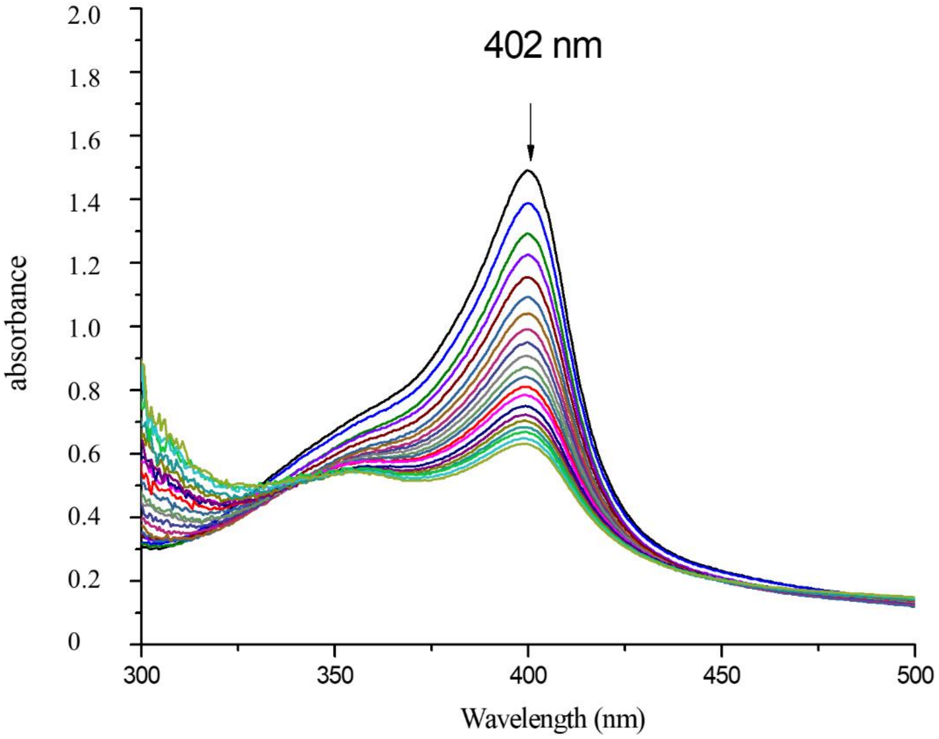

Figure S36- Titration of ferriprotoporphyrin IX [Fe(III)-PPIX, hemin]. Arrow indicates the decrease in absorbance upon increasing concentration of compounds [Ag(ATV)(PPh_3)$_2$] (**1**)

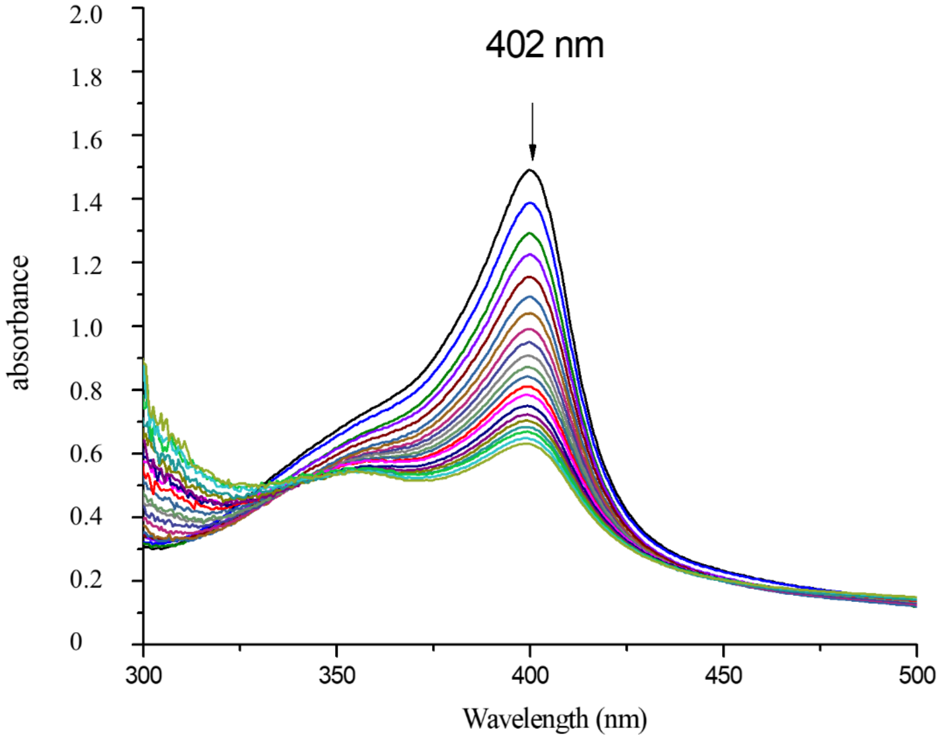

Figure S37- Titration of ferriprotoporphyrin IX [Fe(III)-PPIX, hemin]. Arrow indicates the decrease in absorbance upon increasing concentration of compounds [Au(ATV)(PPh_3)].$2H_2O$ (**2**)

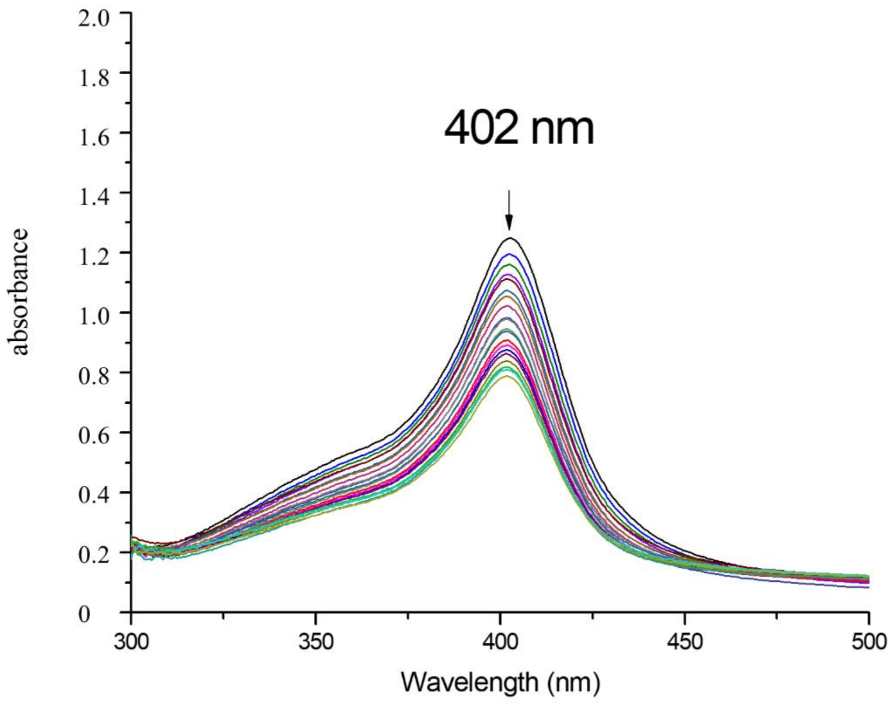

Figure S38- Titration of ferriprotoporphyrin IX [Fe(III)-PPIX, hemin]. Arrow indicates the decrease in absorbance upon increasing concentration of compounds [Cu(ATV)(PPh_3)$_2$] (**3**)

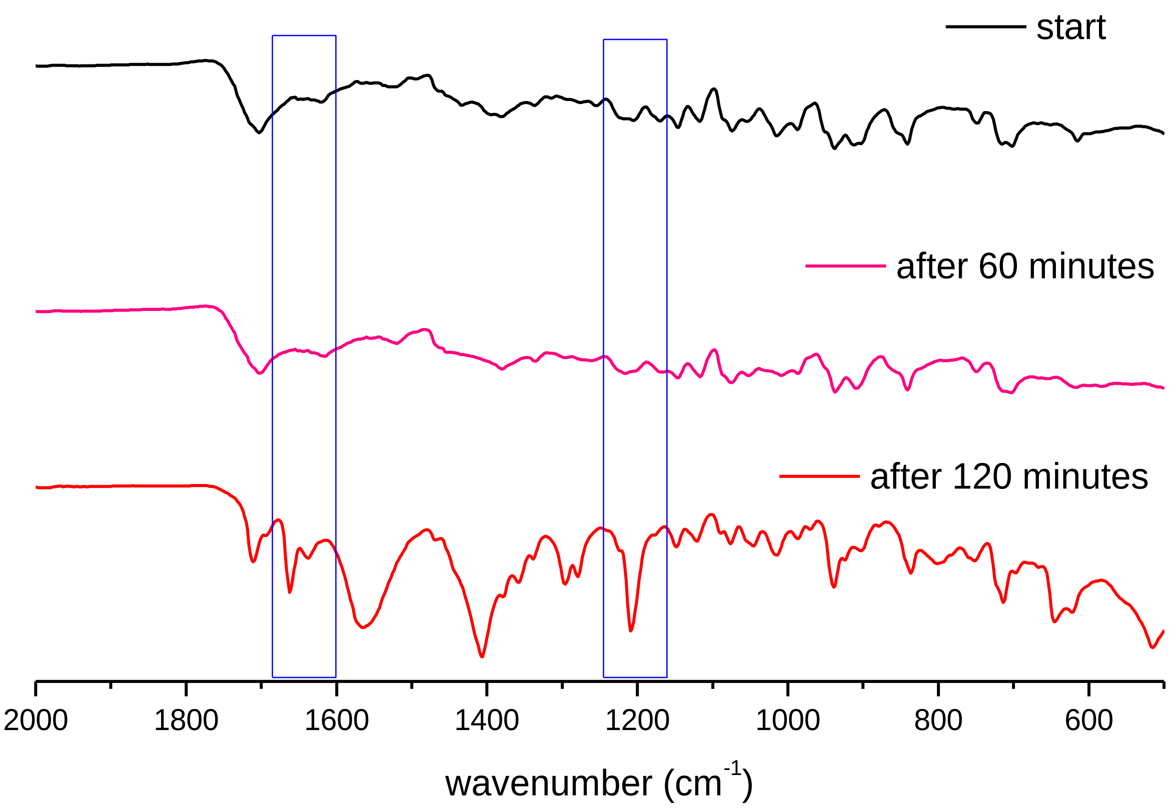

Figure S39- Spectra ß-hematin formation.

References.

1. Hahn, T.; Shmueli, U.; Arthur, J.C.; Wilson (Ed.).; International Tables for Crystallography. *Dordrecht: Reidel.* **1983.** Doi:10.1107/97809553602060000100.

2. Spek, A.L.J.; Single-crystal structure validation. *J. Appl. Cryst*. **2003,** 36 (1), 7-13,
